# Tumor‐Derived Interleukin 35 Promotes Fibrosis in the Tumor Microenvironment of Pancreatic Cancer by Activating Pancreatic Stellate Cells

**DOI:** 10.1002/advs.202509074

**Published:** 2025-11-14

**Authors:** Hui Li, Huizhi Sun, Jing Liu, Yan Wu, Lin Wei, Jianming Li, Yudong Yuan, Peng Xie, Chao Xu, Guolu Luo, Yuqi Guan, Yukuan Feng, Antao Chang, Jihui Hao, Chongbiao Huang

**Affiliations:** ^1^ Pancreas Center, Tianjin Medical University Cancer Institute and Hospital, National Clinical Research Center for Cancer, National Key Laboratory of Draggability Evaluation and Systematic Translational Medicine, Tianjin Key Laboratory of Digestive Cancer Tianjin's Clinical Research Center for Cancer Tianjin 300060 P. R. China; ^2^ Department of Anesthesia Tianjin Medical University Cancer Institute and Hospital Tianjin 300060 P. R. China; ^3^ Department of Pulmonary Oncology Tianjin Medical University Cancer Institute and Hospital Tianjin 300060 P. R. China; ^4^ Department of Breast Surgery Fudan University Shanghai Cancer Center Shanghai 200032 P. R. China; ^5^ Key laboratory of Carcinogenesis and Translational Research (Ministry of Education/Beijing), Department of Pathology Peking University Cancer Hospital & Institute Beijing 100142 P. R. China; ^6^ National Clinical Research Center for Cancer, Key Laboratory of Breast Cancer Prevention and Therapy, Tianjin Medical University, Ministry of Education Cancer Hospital Airport Hospital Tianjin 300000 P. R. China

**Keywords:** fibrosis, PDAC, PSC, tumor‐derived IL‐35

## Abstract

Severe fibrosis, predominantly driven by the activation of pancreatic stellate cells (PSCs), plays a crucial role in the poor prognosis associated with pancreatic ductal adenocarcinoma (PDAC). Understanding the intricate interplay between tumor cells and their microenvironment is essential for deciphering the regulatory mechanisms underlying PSC activation. This study sheds light on the critical role of tumor‐derived interleukin‐35 (IL‐35) in modulating PSC activation, thereby unveiling a promising therapeutic target for mitigating PDAC progression. This study demonstrates that IL‐35, secreted by PDAC cells, serves as a key mediator of bidirectional communication between PDAC cells and PSCs, exacerbating fibrosis. Specifically, IL‐35 upregulates the expression of IGFBP2 and Tsp‐1 in PDAC cells, which subsequently activates PSCs through the IGF‐1R/phosphoinositide 3‐kinase/Akt and transforming growth factor beta signaling pathways, respectively. This sequential activation fosters an environment conducive to tumor cell proliferation and migration, ultimately driving accelerated tumor growth. Collectively, these findings indicate that IL‐35 is a promising therapeutic target whose blockade not only suppresses PSC activation and stromal fibrosis, but also enhances the efficacy of standard chemotherapies (gemcitabine and gemcitabine/nab‐paclitaxel). This provides a strong rationale for its clinical development as a combinatorial strategy in PDAC treatment.

## Graphical abstract

This study demonstrates that tumor‐derived IL‐35 promotes pancreatic cancer fibrosis by indirectly activating pancreatic stellate cells through IGFBP2/IGF‐1R/PI3K‐Akt and Tsp‐1/TGF‐β pathways. IL‐35 blockade reduces stromal fibrosis, restores chemosensitivity, and synergizes with gemcitabine‐based regimens, highlighting IL‐35 as a promising therapeutic target and biomarker for combination therapy in pancreatic ductal adenocarcinoma.

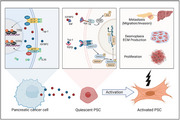

## 1 Introduction

Pancreatic ductal adenocarcinoma (PDAC) is a deadly disease that is resistant to chemotherapy and difficulty to detect early on.^[^ ^1^ ^]^ Severe fibrosis is a hallmark of the tumor microenvironment (TME) of PDAC, contributing significantly to treatment resistance and the malignant behaviors of PDAC cells. Fibrosis creates a barrier that limits the penetration and efficacy of therapeutic drugs, dramatically limiting the clinical benefits of these treatments.^[^ ^2^, ^3^ ^]^ Additionally, fibrous components, such as collagen, act as an extracellular matrix (ECM) that promotes the proliferation, immunosuppression, and migration of PDAC cells by activating the integrins and discoidin domain receptor signaling pathways.^[^ ^4^, ^5^ ^]^ However, the mechanisms through which PDAC cells interact with stromal cells to form a fibrotic TME remain unclear.

Pancreatic stellate cells (PSCs) are integral components of the PDAC stroma.^[^ ^6^ ^]^ Once activated, PSCs exhibit increased proliferation and migration, which play crucial roles in fibrosis formation in PDAC.^[^ ^7^, ^8^ ^]^ Various signaling pathways have been identified as triggers for PSC activation, including the phosphoinositide 3‐kinase (PI3K) pathway,^[^ ^9^ ^]^ transforming growth factor beta (TGF‐β) pathway,^[^ ^10^ ^]^ and mitogen‐activated protein kinase (MAPK) pathway.^[^ ^11^ ^]^ Additionally, specific molecules such as angiotensin,^[^ ^12^ ^]^ galectin‐1,^[^ ^13^ ^]^ endothelin‐1^[^ ^14^ ^]^ have also been implicated in the activation of PSCs. Numerous studies have revealed the bidirectional interactions between PSCs and PDAC cells. PSCs educated by PDAC cells display increased proliferation, migration, and enhanced synthesis of the ECM.^[^ ^15^ ^]^ In turn, activated PSCs (aPSCs) markedly facilitate the malignant behaviors of PDAC cells by promoting proliferation and inducing angiogenesis, immune evasion, epithelial‐mesenchymal transition (EMT), migration, and treatment resistance.^[^ ^16^ ^]^ Therefore, exploring interventions that disrupt the interaction between tumor cells and PSCs within the TME may unveil new therapeutic strategies for inhibiting PDAC progression.

Interleukin (IL)‐35, a heterodimer comprising of IL‐12A (p35) and EBI3 subunits, belongs to the IL‐12 family.^[^ ^17^ ^]^ Unlike other members of this family, IL‐35 possesses immunosuppressive properties by expanding regulatory T cells (Tregs) and regulatory B cells, while inhibiting the function of effector T cells, Th1 cells, Th17 cells, and macrophages.^[^ ^17^, ^18^ ^]^ Consequently, IL‐35 has been recognized as an important factor in tumor progression.^[^ ^19^, ^20^ ^]^ Our previous studies demonstrated that IL‐35 is highly expressed in PDAC cells, facilitating tumor metastasis and angiogenesis, thereby contributing to tumor progression.^[^ ^21^, ^22^ ^]^ However, the specific effects of IL‐35 in PDAC fibrosis remain unknown.

In the present study, our findings indicate that tumor‐derived IL‐35 plays a pivotal role in PSC activation and fibrosis formation in PDAC. IL‐35 induces the secretion of IGFBP2 and Tsp‐1 in PDAC cells, subsequently triggering the activation of PSCs, which, in turn, facilitates tumor growth and metastasis. Moreover, treatment with an IL‐35 neutralizing antibody effectively inhibited PDAC fibrosis and enhanced the therapeutic efficacy of the conventional chemotherapeutic drug gemcitabine (GEM). Taken together, our study revealed new roles and mechanisms of IL‐35 in promoting PDAC progression and offers a new combination strategy for the treatment of PDAC.

## 2 Results

### 2.1 Tumor‐derived IL‐35 Levels are Positively Correlated with Fibrosis in the TME of Pancreatic Cancer

**Figure 1:**
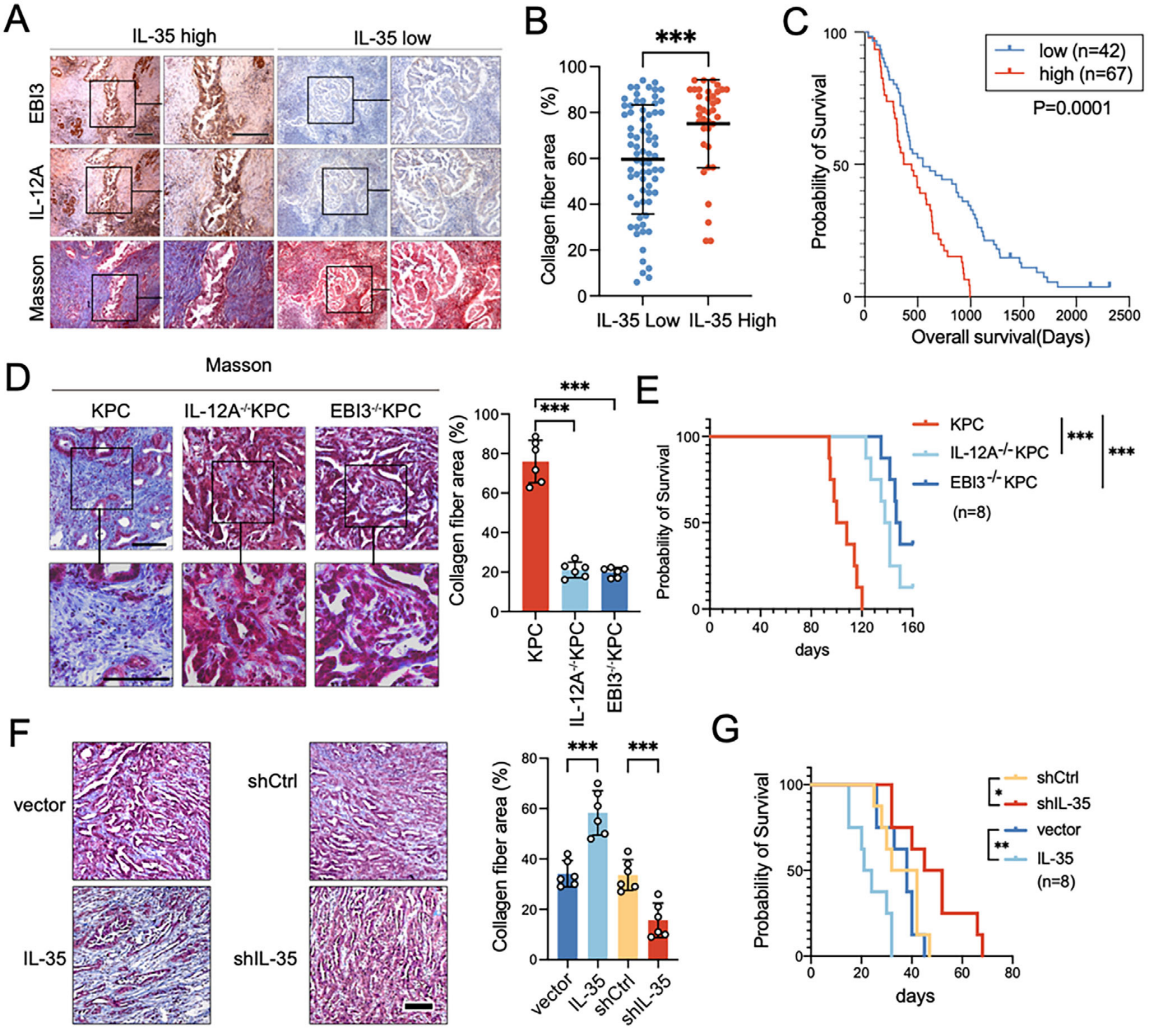
Tumor‐derived IL‐35 promotes fibrosis in PDAC. A) Representative images of IHC staining for IL‐35 and Masson's trichrome staining for collagen fibers (collagen stained blue). B) Distribution of the percentage area occupied by blue‐stained collagen in 109 PDAC slices with different levels of IL‐35 expression. Data were analyzed using unpaired t‐test. C) Kaplan‐Meier analysis of the overall survival of 109 PDAC patients according to different Masson staining scoring. Collagen fiber area exceeding 50% was categorized as high (n = 67), Collagen fiber area of 50% or less was categorized as low (n = 42).D) Left, representative images of Masson trichrome staining in KPC, IL12A^−/−^KPC, and EBI3^−/−^KPC mice (tumor diameter was ≈8 mm). Right, the percentage of collagen fiber area to the total pancreas in indicated mice was shown. Data were analyzed using unpaired t‐test. E) Kaplan‐Meier analysis comparing the survival of indicate group, the observation endpoint is set at 160 days postnatally (n=8). F) 1x10^5^ indicated KPC cells were orthotopically injected into the pancreas of 5‐week‐old SCID mice (n = 6) to induce tumor formation. After 30 days, harvest the tumors. Representative images of Masson trichrome staining and the percentage of collagen fiber area to the total pancreas in the indicated group are shown. Data were analyzed using unpaired t‐test. G) Kaplan‐Meier analysis comparing the survival of indicate group (n = 8). Shown are mean ± SD; *p <.05. **p <.01. ***p <.001. Scale bars,100 µm.

In the early stages of our research on IL‐35, we noted that patients with PDAC expressing higher levels of IL‐35 also showed increased degrees of fibrosis in the TME compared to those expressing lower levels of IL‐35. To explore the potential role of IL‐35 in fibrosis, we collected 109 surgical samples from patients with resectable PDAC and performed immunohistochemical (IHC) and Masson's trichrome staining to assess the relationship between tumor IL‐35 expression and fibrosis. The results showed a significant increase in fibrotic areas in tumor tissues with high IL‐35 expression compared to those with low IL‐35 expression (**Figure** **1**A,B). These findings suggest a positive correlation between IL‐35 expression and fibrosis in PDAC. Additionally, patients with high levels of fibrosis had a poorer prognosis (*p* < 0.0001; Figure 1C).

To further validate the effect of IL‐35 on PDAC fibrosis, we generated two KPC (LSL‐Kras^G12D/+^‐Trp53^R172H/+^‐Pdx1‐Cre) mouse models with conditional knock‐out of IL‐12A (IL‐12A^flox/flox^ KPC) or EBI3 (EBI3^flox/flox^ KPC) (Figure S1A,B, Supporting Information), the two subunits of IL‐35.

Ultrasound monitoring revealed that knockout of either IL‐12A or EBI3 significantly reduced the size of spontaneous tumors compared to wild‐type KPC mice of the same age, indicating that IL‐35 plays an important role in tumorigenesis and tumor growth (Figure S1C, Supporting Information). Moreover, Masson's trichrome staining of spontaneous tumors of similar size also indicated that knockout of IL‐35 notably inhibited the fibrosis of PDAC in KPC mouse models (Figure 1D). Moreover, the knockout of IL‐12A or EBI3 significantly increased the survival of KPC mice (Figure 1E). We constructed pancreatic orthotopic tumor models using KPC‐derived cell lines with IL‐35 overexpression, knockdown, or plasmid control in severe combined immunodeficiency (SCID) mice. Forced IL‐35 expression significantly promoted tumor growth (Figure S1D,E, Supporting Information) and fibrosis (Figure 1F), leading to shorter survival times in tumor‐bearing mice (Figure 1G). Conversely, IL‐35 knockdown markedly reduced tumor growth and fibrosis, resulting in a more favorable prognosis (Figure 1F,G; Figure S1E, Supporting Information). These findings provide strong evidence that IL‐35 plays a crucial role in fibrosis formation in PDAC.

### 2.2 Tumor‐derived IL‐35 Induces Activation of PSCs

Since PSCs are one of the major cell types responsible for fibrosis in the TME of PDAC,^[^ ^23^, ^24^ ^]^ we hypothesized that tumor‐derived IL‐35 might enhance PSC activation, thus inducing fibrosis. To test this, we conducted IHC staining on surgical PDAC samples to evaluate the expression levels of IL‐35, α‐smooth muscle actin (α‐SMA), and collagen type 1 (COL‐1). Both α‐SMA and COL‐1 are markers of aPSCs. The results revealed a significant positive correlation among IL‐35, α‐SMA, and COL‐1 in PDAC tissues (Figure S2A,B, Supporting Information). Additionally, multiplex fluorescent IHC staining of allograft tumors derived from KPC cell lines with IL‐35 overexpression, knockdown, or plasmid control indicated that IL‐35 overexpression increased α‐SMA and COL‐1 levels, while IL‐35 knockdown reduced their expression (Figure S2C, Supporting Information). Consistent results were obtained from spontaneous tumors with a conditional IL‐35 knockout, demonstrating that tumor‐derived IL‐35 is necessary for PSC activation (Figure S2D, Supporting Information).

To further investigate the potential role of IL‐35 in driving phenotypic heterogeneity among PSCs, we reanalyzed publicly available single‐cell RNA sequencing datasets from PDAC tumors,^[^ ^25^ ^]^ with a specific focus on PSC subpopulations. Unsupervised clustering analysis revealed three dominant PSC subtypes: myofibroblastic (myCAF), inflammatory (iCAF), and antigen‐presenting (apCAF) phenotypes. Tumors exhibiting high IL‐35 expression showed a significantly increased proportion of myCAF‐like PSCs compared with other subtypes (*p* < 0.05), suggesting that IL‐35 signaling may preferentially promote a myofibroblastic phenotype, which is closely associated with ECM deposition and fibrotic processes (Figure S3, Supporting Information).

**Figure 2:**
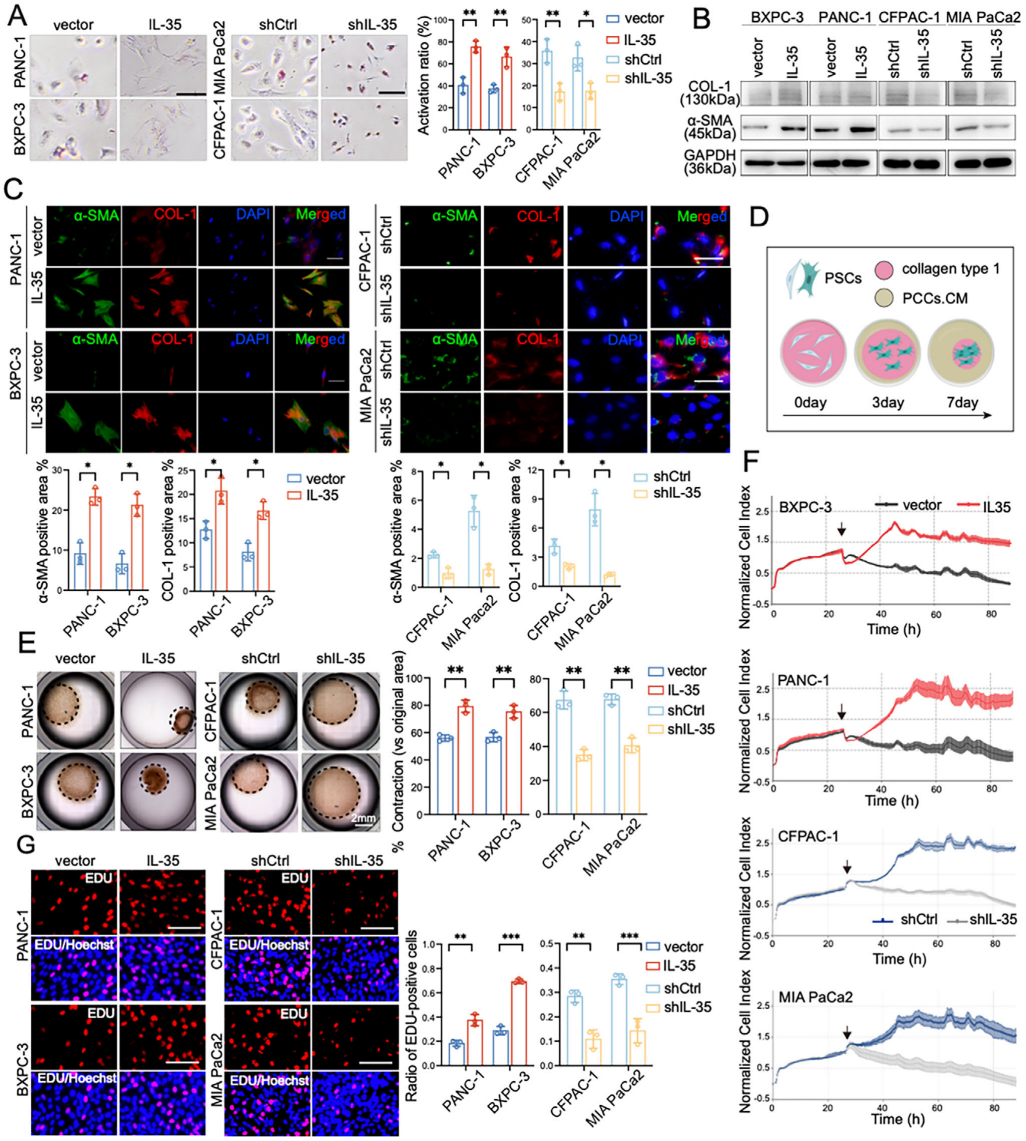
Increased IL‐35 expression contributes dramatically to the activation of PSCs. Supernatants from indicated PDAC cells with or without IL‐35 up‐regulated/down‐regulated were used as the conditioned media (CM), the isolated human primary PSCs were treated with different CM for 72h. A) Representative images of oil red o staining of PSC were shown, and the activation ratio was counted. B) Western blotting was used to detect the activation marker of PSCs. C) Representative images of immunofluorescence staining of activation markers in PSCs. D) Schematics of collagen contraction assay. E) Collagen gel containing primary PSCs were treated with different CM. Representative collagen gel images are shown (7 Day). F) PSCs of the indicated group were detected by Real‐Time CelI AnaIysis (RTCA) system. G) Representative images of Edu proliferation analysis in the indicated groups. Data were analyzed using unpaired t‐test. Experiments above were repeated three times independently. Shown are mean ± SD; * *p* <.05. **p <.01.***p <.001. Scale bars, 100 µm.

To validate these findings, we isolated quiescent human and murine PSCs using a well‐established method and treated them with conditioned medium (CM) from PDAC cell lines with IL‐35 overexpression, knockdown, or a plasmid control to assess PSC activation.^[^ ^7^, ^26^ ^]^ The PDAC cell lines used in this study were established as described in our prior research.^[^ ^21^ ^]^ As anticipated, treatment with CM from IL‐35 overexpressing PDAC cell lines significantly promoted the activation of quiescent human and murine PSCs, as indicated by a significant decrease in intracellular lipid droplets observed in Oil Red staining (**Figure** **2**A), induction of stellate cell morphology (Figure 2A; Figure S4A, Supporting Information), and up‐regulation of α‐SMA and COL‐1 revealed by immunoblotting and immunofluorescent staining (Figure 2B,C; Figure S4B,C, Supporting Information). Conversely, co‐culture with CM from IL‐35 knockdown PDAC cell lines markedly inhibited PSC activation (Figure 2A–C; Figure S4B,C, Supporting Information). Functional assays further indicated that the CM from IL‐35 overexpressing PDAC cells enhanced collagen contraction (Figure 2D,E; Figure S4D, Supporting Information), cell viability (Figure 2F), and EdU incorporation (Figure 2G) into quiescent PSCs. In contrast, CM from IL‐35 knockdown PDAC cells suppressed PSC capabilities in proliferation (Figure 2D–G; Figure S4D, Supporting Information). Collectively, these results demonstrated that tumor‐derived IL‐35 mediates PSC activation, leading to fibrosis in the PDAC tumor microenvironment.

aPSCs have been shown to play pivotal roles in tumor progression, including proliferation, invasion, immunosuppression, and drug resistance.^[^ ^27^ ^]^ Consistent with these findings, the treatment of PDAC cells with CM from aPSCs, obtained through co‐culture with CM from IL‐35 overexpressing PDAC cell lines, enhanced the migration, invasiveness, and proliferation of tumor cells in vitro (Figure S5A–D, Supporting Information), and promoted allograft tumor growth in pancreatic orthotopic tumor models^[^ ^2^ ^]^ (Figure S5E, Supporting Information). These observations indicate that tumor‐derived IL‐35 induces PSC activation, thereby facilitating PDAC progression.

### 2.3 IGFBP2 and Tsp‐1 are Essential for IL‐35‐induced PSC Activation

**Figure 3:**
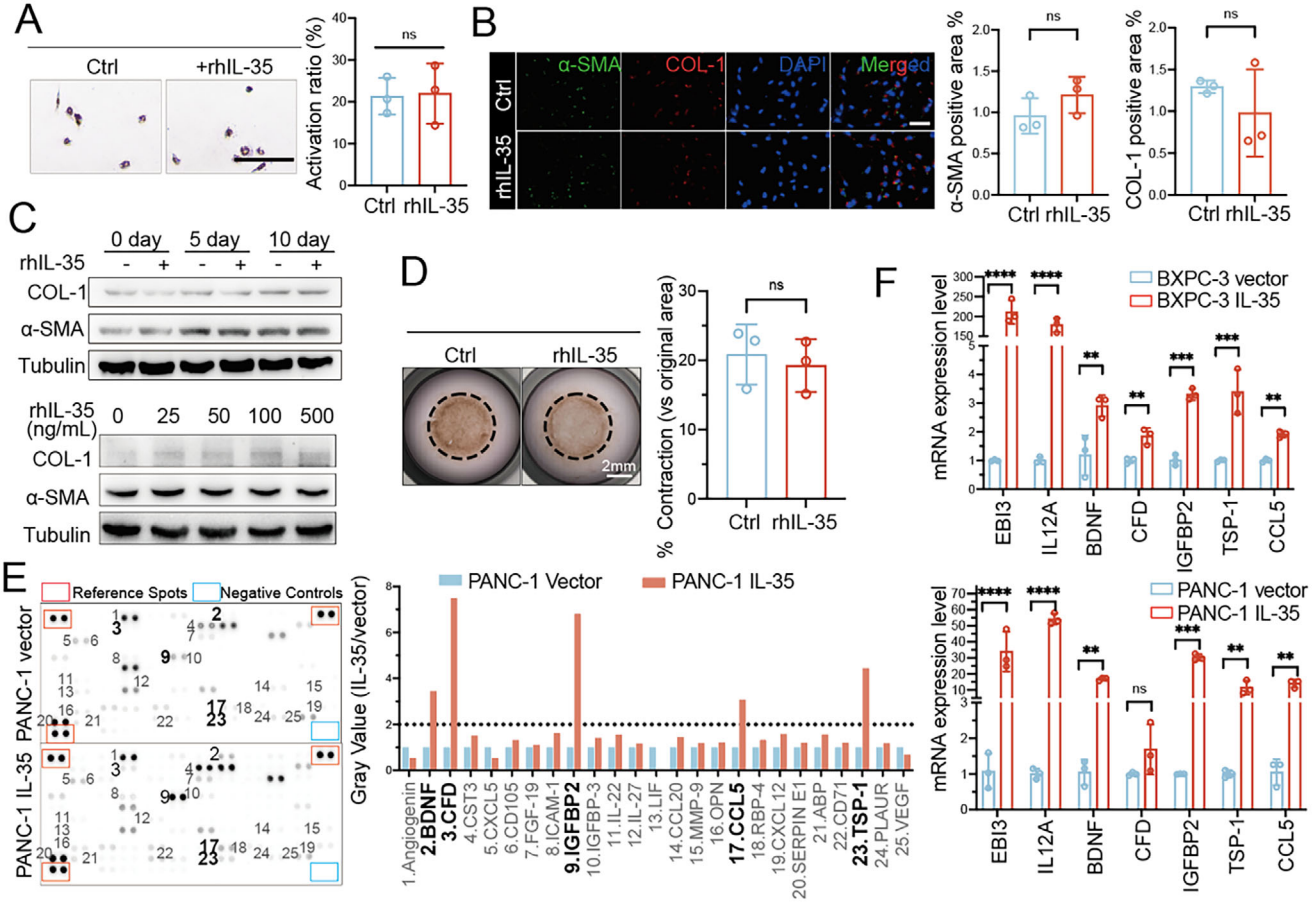
IL‐35 failed to directly activate the PSCs. The isolated human primary PSCs were treated with or without IL‐35 recombinant protein (rhIL‐35) (100 ng mL^−1^) for 72 h. A) Representative images of oil red o staining of PSCs were shown, and the activation ratio was counted. B) Representative images of immunofluorescence staining of activation markers in PSCs. C) Western blotting was used to detect the activation marker of PSCs for different time intervals or varying dosage conditions. D) Collagen gel containing primary PSCs were treated with or without rhIL‐35 for 7 days. Representative collagen gel images are shown. E) Representative dot blots of supernatants from indicated PANC‐1 cells with or without IL‐35 up‐regulated with 105 cytokine arrays. Corresponding quantitation of fold change in BDNF, CFD, IGFBP2, CCL5, and Tsp‐1 signals derived from protein arrays. F) qRT‐PCR analysis of BDNF, CFD, IGFBP2, CCL5, and Tsp‐1 mRNA of PDAC cells with or without IL‐35 overexpression. Data were analyzed using unpaired t‐test. Experiments above were repeated three times independently. Shown are mean ± SD; *p <.05. **p <.01. *** *p* <.001. **** *p* <.0001. ns no significance. Scale bars, 100 µm.

**Figure 4:**
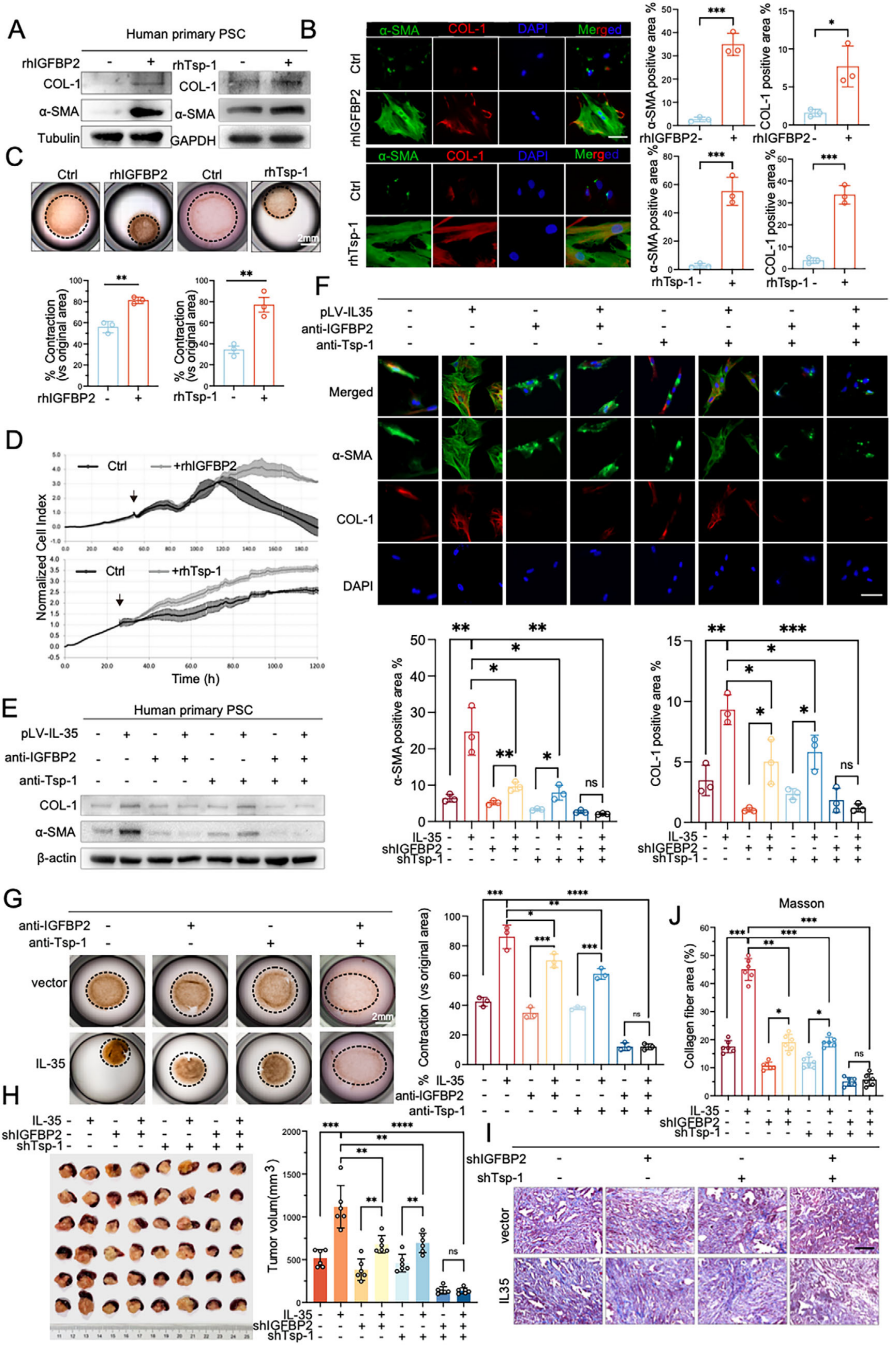
IGFBP2 and Tsp‐1 are essential for IL‐35‐induced PSC activation. A–D) Human primary PSCs were treated with or without the recombinant proteins IGFBP2/Tsp‐1 (rhIGFBP2/Tsp‐1) (100 ng mL^−1^) for A,B) 72 h or C) 7 days. A) The activation marker of PSCs was detected using Western blotting, B) immunofluorescence staining, and C) representative collagen gel images are displayed. D) The cell index of PSCs was analyzed using RTCA. E–G) Supernatants from PANC‐1 cells with or without IL‐35 up‐regulated were used as the CM. Then, the CM was treated with IGFBP2 (50 ng mL^−1^) or Tsp‐1 neutralizing antibodies (100 ng mL^−1^) for 1 h, followed by co‐culturing with primary PSCs for E,F) 72 h or G) 7 days. E) The activation marker of PSCs was detected using Western blotting. F) Immunofluorescence staining, and G) representative collagen gel images were displayed. Experiments above were repeated three times independently. H–J) Engineering modifications were performed on KPC cell lines with or without overexpressing IL‐35 to downregulate IGFBP2 or Tsp‐1. Then, 1 × 10^5^ indicated cells were orthotopically injected into the pancreas of 5‐week‐old SCID mice. After 30 days, harvest the tumors. Photos and tumor volume of primary tumors were shown (H). Representative images of Masson trichrome and percentage of collagen fiber area to the total pancreas in indicate mice were shown (I,J). Data were analyzed using unpaired t‐test. Shown are mean ± SD; * *p* <.05. ** *p* <.01. *** *p* <.001. **** *p* <.0001. ns no significance. Scale bars, 100 µm.

To investigate the mechanism by which IL‐35 mediates PSCs activation, we treated human quiescent human PSCs with recombinant human IL‐35 protein (rhIL‐35) and performed various assays, including Oil Red O staining, immunofluorescence, immunoblotting, and collagen contraction. Interestingly, rhIL‐35 treatment did not activate PSCs (**Figure** **3**A–D), suggesting that it may indirectly mediate PSCs activation. Further research revealed that quiescent PSCs express minimal levels of IL‐35 receptors GP130 and IL12Rβ2 compared to tumor cells (Figure S6A, Supporting Information). Given that the CM from IL‐35‐overexpressing PDAC cells effectively activates PSCs, we hypothesized that IL‐35 facilitates the expression of other secreted factors that directly induce PSC activation. A dot blot assay identified enrichment of BDNF, CCL5, CFD, IGFBP2, and Tsp‐1 in the CM from IL‐35‐overexpressing PANC‐1 cells compared to the control cells (Figure 3E). Additionally, quantitative real‐time PCR (qRT‐PCR) showed a significant increase in the mRNA levels of these cytokines in BXPC‐3 and PANC‐1 cells overexpressing IL‐35 (Figure 3F). Recombinant human IGFBP2 (rhIGFBP2, 100 ng mL^−1^) and Tsp‐1 (rhTsp‐1, 100 ng mL^−1^), but not BDNF (rhBDNF), CCL5 (rhCCL5), or CFD (rhCFD), effectively induced α‐SMA and COL‐1 expression (**Figure** **4A,B**; Figure S6B,C, Supporting Information) and reduced intracellular lipid droplets in PSCs (Figure S7A, Supporting Information), leading to PSC activation.

In a co‐culture model of human quiescent PSCs with CM from IL‐35‐overexpressing PANC‐1 cells, neutralization of IGFBP2 or Tsp‐1 alone partially impeded IL‐35‐mediated PSC activation, whereas simultaneous neutralization of both IGFBP2 and Tsp‐1 completely abrogated the effect of IL‐35 on PSC activation (Figure 4E–G; Figure S7B,C, Supporting Information). These results were further supported by knockdown experiments using lentivirus‐packaged shRNAs targeting *IGFBP2* and *THBS1* (Figure S8A,B, Supporting Information). Simultaneous knockdown of *IGFBP2* and *THBS1* in KPC cells completely abolished IL‐35‐induced PSC activation in cell co‐culture models (Figure S8C,D, Supporting Information), as well as tumor growth and fibrosis in pancreatic orthotopic tumor models (Figure 4H–J). These findings demonstrated that IGFBP2 and Tsp‐1 serve as downstream effectors of IL‐35 in mediating PSC activation.

### 2.4 IL‐35 Induces IGFBP2 and Tsp‐1 Expression via the STAT Signaling Pathway

**Figure 5:**
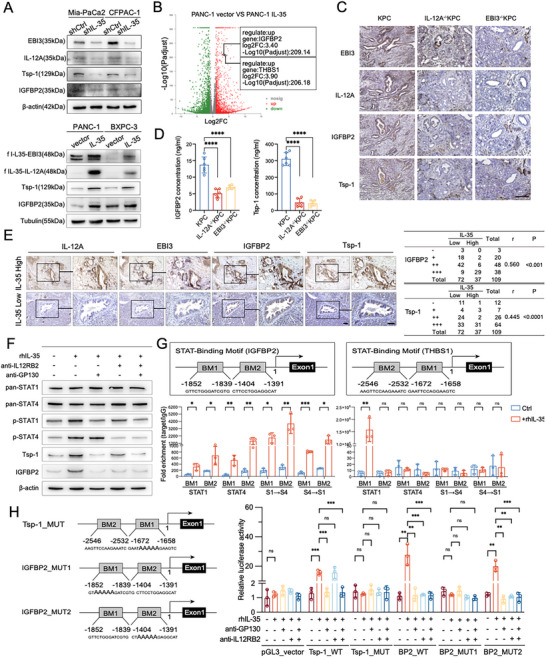
Molecular mechanism of IL‐35 transcriptional regulation of IGFBP2 and Tsp‐1. A) Western blotting was performed on EBI3, IL‐12A, IGFBP2, and Tsp‐1 in the indicated cell lines. B) Genome‐wide mRNA sequencing was conducted to compare the expression profiles between PANC‐1 cells with or without IL‐35 overexpression. The genes significantly altered by IL‐35 upregulation (|log2FC > 2|) are presented in a volcano plot. C) Representative images of IHC staining targeted on IL‐12A, EBI3, IGFBP2, and Tsp‐1 in PDAC tissues of KPC, IL‐12A^−/−^KPC and EBI3^−/−^KPC mice. D) ELISA was used to measure the levels of IGFBP2 and Tsp‐1 in the peripheral blood of 4‐month‐old KPC, IL‐12A^−/−^KPC, and EBI3^−/−^KPC mice. E) Representative images of IHC staining targeted on IL‐12A, EBI3, IGFBP2, and Tsp‐1 in human PDAC tissues, and statistical analysis of IHC results of IGFBP2/Tsp‐1 and IL‐35 expression in 109 human PDAC surgical samples in the table. Data were analyzed using Spearman correlation analysis. F) PANC‐1 cells were pretreated with or without anti‐GP130 antibody (100 ng mL^−1^) and/or anti‐IL12RB2 antibody (2 µg mL^−1^) for 30 min, and then, rhIL‐35 (100 ng mL^−1^) was added to culture for another 16 h. The cells were subjected to western blotting assays. G) Human IGFBP2 and THBS1 promoter, and two of the identified STAT binding motifs were investigated. CHIP analysis of PANC‐1 cells pretreated with or without rhIL‐35 (100 ng mL^−1^) for 90 min. Chromatin was immunoprecipitated with anti‐STAT1 or anti‐STAT4 antibodies and then subjected to RT‐PCR analysis. Then, CHIP‐reCHIP analysis of PANC‐1 cells prepared as in above, chromatin was first immunoprecipitated with anti‐STAT1 or anti‐STAT4 antibody and then immunoprecipitated again with anti‐STAT4 (S1→S4) or anti‐STAT1 (S4→S1). H) Left, Schematic of the binding motifs mutation in IGFBP2 and THBS1 promoter. Right, Dual luciferase activity of reporter plasmids with the wild‐type or mutation of IGFBP2/THBS1 fused to the luciferase gene following IL‐35 co‐transfection in HEK293T cells. Data were analyzed using unpaired t‐test. Experiments above were repeated three times independently. Shown are mean ± SD; *p <.05. **p <.01. ***p <.001. ****p <.0001. ns no significance. Scale bars,100 µm.

Our study utilized various techniques, including immunoblotting, RNA sequencing (RNA‐seq), qRT‐PCR, and ELISA assays to investigate the impact of IL‐35 on IGFBP2 and Tsp‐1 expression. We found that forced expression of IL‐35 significantly enhanced both mRNA and protein levels as well as the secretion of IGFBP2 and Tsp‐1 in PANC‐1 and BXPC‐3 cells. Conversely, knockdown of IL‐35 notably inhibited IGFBP2 and Tsp‐1 expression and secretion in Mia‐PaCa2 and CFPAC‐1 cells (**Figure** **5**A,B; Figure S9A,B, Supporting Information). In the pancreatic tumors and peripheral blood of IL‐12A^−/−^KPC and EBI3^−/−^KPC mice, the levels of IGFBP2 and Tsp‐1 were also significantly lower than those in KPC mice (Figure 5C,D). IHC staining of serial sections of surgical PDAC samples also revealed a significant positive correlation between IL‐35 expression and IGFBP2 or Tsp‐1 levels (Figure 5E). Collectively, these results suggest that IL‐35 promotes the expression and secretion of IGFBP2 and Tsp‐1, primarily at the mRNA level.

IL‐35 signaling is mediated by binding to its receptor, a homodimer or heterodimer composed of IL12Rβ2 and GP130, which subsequently activates the JAK‐STAT signaling pathway. In PANC‐1 cells, inhibiting IL12Rβ2 with a blocking antibody impaired STAT4 phosphorylation and effectively suppressed IL‐35‐induced IGFBP2 expression, without affecting Tsp‐1 expression (Figure 5F). In contrast, blocking GP130 hindered STAT1 phosphorylation and completely abolished IL‐35‐induced expression of both IGFBP2 and Tsp‐1, demonstrating an effect comparable to the combined blockade of GP130 and IL12Rβ2 (Figure 5F). These results suggest that IL‐35 induces IGFBP2 expression by binding to the IL12Rβ2:GP130 heterodimer and triggers Tsp‐1 expression, activating the GP130:GP130 homodimer in PDAC cells.

According to the JASPAR database, there are two predicted binding sites for STAT1/4 in the promoter regions of both *IGFBP2* and *THBS1* (the gene encoding Tsp‐1). Chromatin immunoprecipitation (ChIP) and ChIP‐reChIP analyses were performed to precisely identify the binding sites and patterns of STAT1 and STAT4 on the *IGFBP2* and *THBS1* promoters. Our data indicated that IL‐35 induces the formation of the pSTAT1:pSTAT4 heterodimer, which subsequently binds to the two binding sites on the *IGFBP2* promoter, while also promoting the binding of the pSTAT1:pSTAT1 homodimer to binding site 1 on the *THBS1* promoter (Figure 5G). We then mutated these binding sites to disrupt their interaction with the STAT transcription factors and conducted dual‐luciferase reporter assays to determine whether these sites are responsible for the IL‐35‐induced transcriptional activation of *IGFBP2* and *THBS1*. Notably, IL‐35‐induced transcriptional activation of *THBS1* was impeded by mutation of the binding site 1 on the promoter, and by blocking of GP130, but not IL12Rβ2. On the other hand, mutation of the binding site 1, not binding site 2, completely abolished IL‐35‐induced transcriptional activation of IGFBP2; blocking GP130 or IL12Rβ2 had the same effect (Figure 5H). These findings demonstrate that IL‐35 binds to the IL12Rβ2:GP130 heterodimer, which activates the heterodimerized pSTAT1:pSTAT4 transcription factor to enhance IGFBP2 expression. Simultaneously, IL‐35 binds to the GP130:GP130 homodimer, which activates the cognate pSTAT1:pSTAT1 to induce Tsp‐1 expression in PDAC cells.

### 2.5 IGFBP2 Activates PSCs through the IGF‐1R‐PI3K‐Akt Signaling Pathway, while Tsp‐1 Induces PSC Activation via TGF‐β Signaling

Next, we investigated the mechanism by which IGFBP2 and Tsp‐1 activate PSCs. Five primary cell lines and one immortalized cell line were treated with or without rhIGFBP2, followed by RNA‐seq analysis to identify differentially expressed genes regulated by IGFBP2 (Figure S10A, Supporting Information). Kyoto Encyclopedia of Genes and Genomes pathway enrichment analysis of this gene set revealed significant enrichment of the PI3K‐Akt signaling pathway in PSCs treated with rhIGFBP2 (Figure S10B, Supporting Information). Consistent with this finding, immunoblotting of primary PSCs revealed a notable increase in Akt phosphorylation following treatment with rhIGFBP2 (Figure S10C, Supporting Information). Furthermore, immunofluorescence staining and collagen contraction assays indicated that pretreatment with the PI3K‐Akt inhibitor LY294002 entirely abolished the activation of PSCs induced by rhIGFBP2 (Figure S10D,E, Supporting Information). These results suggest that IGFBP2 activates PSCs via the PI3K‐Akt signaling pathway.

**Figure 6:**
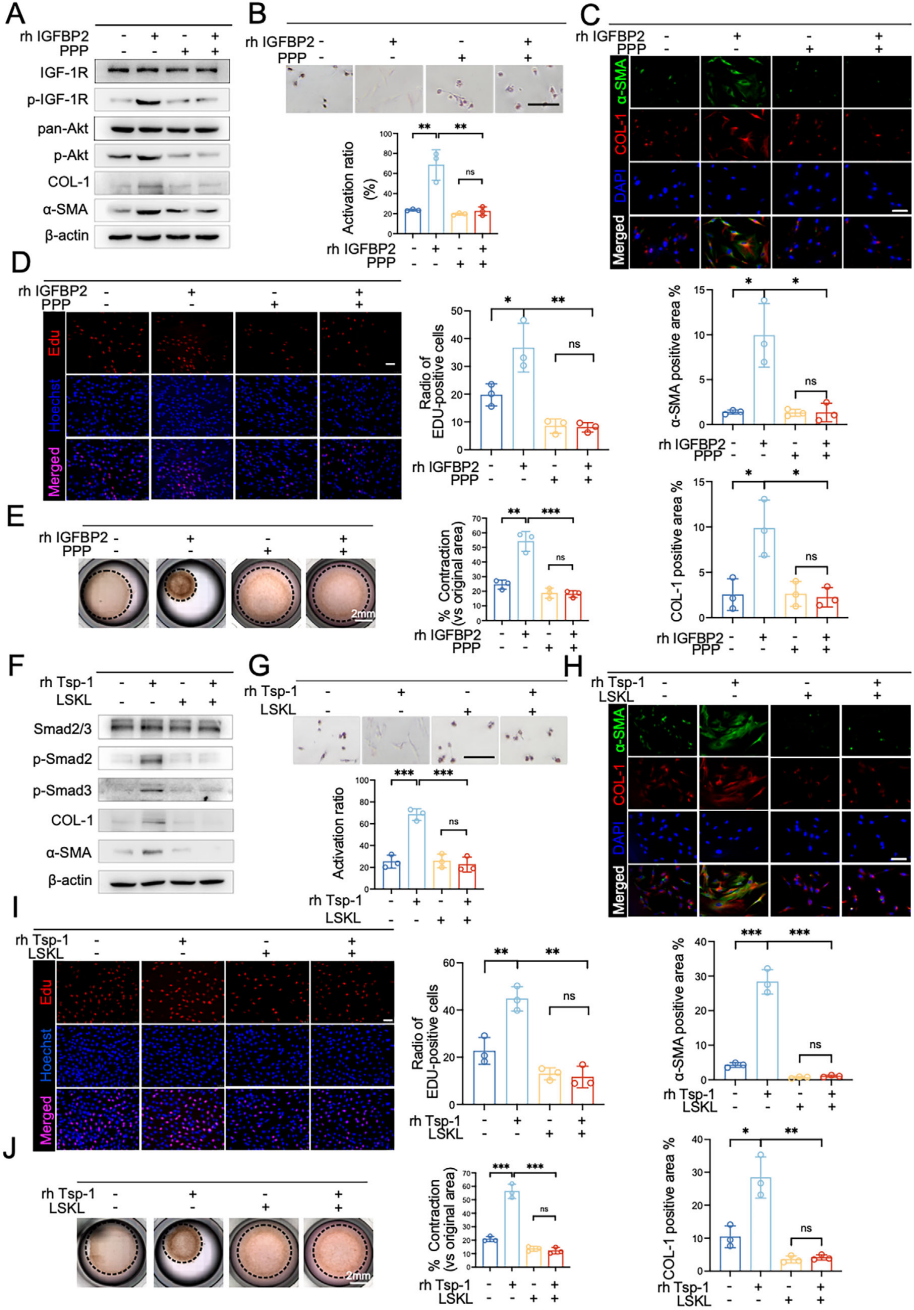
Molecular mechanism of PSCs activation mediated by IGFBP2 and Tsp‐1. A–E) The human primary PSCs were either pretreated with or without 2.5 µm Picropodophyllotoxin (PPP, a selective insulin‐like growth factor‐1 receptor (IGF‐1R) inhibitor) for 2 h, and then, rhIGFBP2 (100 ng mL^−1^) was added to culture for another A–D) 72 h or E) 7 days. The activation of PSCs was detected using A) Western blotting, B) oil red o staining, C) Immunofluorescence staining. D) Representative images of EdU proliferation analysis and E) collagen gel images were displayed, and the data were analyzed using an unpaired t‐test. F–J) The human primary PSCs were either pretreated with or without 0.5 µm LSKL (Inhibitor of Thrombospondin (Tsp‐1)) for 6 h, and then, rhTsp‐1 (100 ng mL^−1^) was added to culture for another F–I) 72 h or J) 7 days. The activation of PSCs was detected using F) Western blotting, G) oil red o staining, H) Immunofluorescence staining.I) Representative images of EdU proliferation analysis and J) collagen gel images were displayed, and the data were analyzed using an unpaired t‐test. Experiments above were repeated three times independently. Shown are mean ± SD; *p <.05. ** *p* <.01. ***p <.001. ****p <.0001. ns no significance. Scale bars, 100 µm.

As a binding protein for insulin‐like growth factors (IGFs), IGFBP2 has been reported to enhance the bioavailability and stability of IGFs, thereby promoting IGF‐1R activation, which results in activation of the PI3K‐Akt pathway.^[^ ^28^ ^]^ Consequently, we hypothesized that IGFBP2 facilitates IGFs‐IGF‐1R signaling, which activates the PI3K‐Akt pathway, leading to PSC activation. To test this hypothesis, quiescence PSCs were pretreated with the IGF‐1R inhibitor picropodophyllin (PPP), followed by stimulation with rhIGFBP2. Subsequent analyses included immunoblotting, Oil Red O staining, immunofluorescence, EdU incorporation, and collagen contraction assays. Notably, blocking of IGF‐1R abrogated the IGFBP2‐induced phosphorylation of IGF‐1R downstream of Akt (**Figure** **6**A), subsequently restricting the PSC activation. This was revealed by an increase in intracellular lipid droplets, a reduction in α‐SMA and COL‐1 expression, decreased proliferation, and reduced collagen contraction (Figure 6B–E). These results demonstrate that IGFBP2 activates PSCs through the IGF‐1R‐PI3K‐Akt signaling cascade.

Tsp‐1 is known for its role in activating the latent TGF‐β1,^[^ ^29^ ^]^ a key mediator of fibrogenesis.^[^ ^30^ ^]^ Analysis of free active TGF‐β1 levels in different cells showed that free active TGF‐β1 levels were elevated in the supernatants of IL‐35‐overexpressing cells and reduced in IL‐35‐silenced cells compared to controls (Figure S11A,B, Supporting Information). To determine whether Tsp‐1 induces PSC activation by activating TGF‐β signaling, we added LSKL, a competitive TGF‐β1 antagonist, to the culture medium containing fetal bovine serum and subsequently treated the samples with rhTsp‐1. We then measured the levels of free active TGF‐β1. The results indicated that LSKL significantly inhibited Tsp‐1‐mediated activation of TGF‐β1 (Figure S11C, Supporting Information). Quiescent PSCs were pretreated with LSKL before exposure to rhTsp‐1. As anticipated, inhibition of TGF‐β activation completely abolished Tsp‐1‐induced phosphorylation of Smad2 and Smad3, two downstream effectors associated with TGF‐β‐mediated fibrogenesis (Figure 6F). This blockage completely impeded the activation of PSCs stimulated by Tsp‐1 (Figure 6F–J). Therefore, these findings indicate that Tsp‐1 activates PSCs through the TGF‐β signaling pathway.

### 2.6 Targeting IL‐35 Mitigates Fibrosis in PDAC and Enhances Responsiveness to GEM Therapy

**Figure 7:**
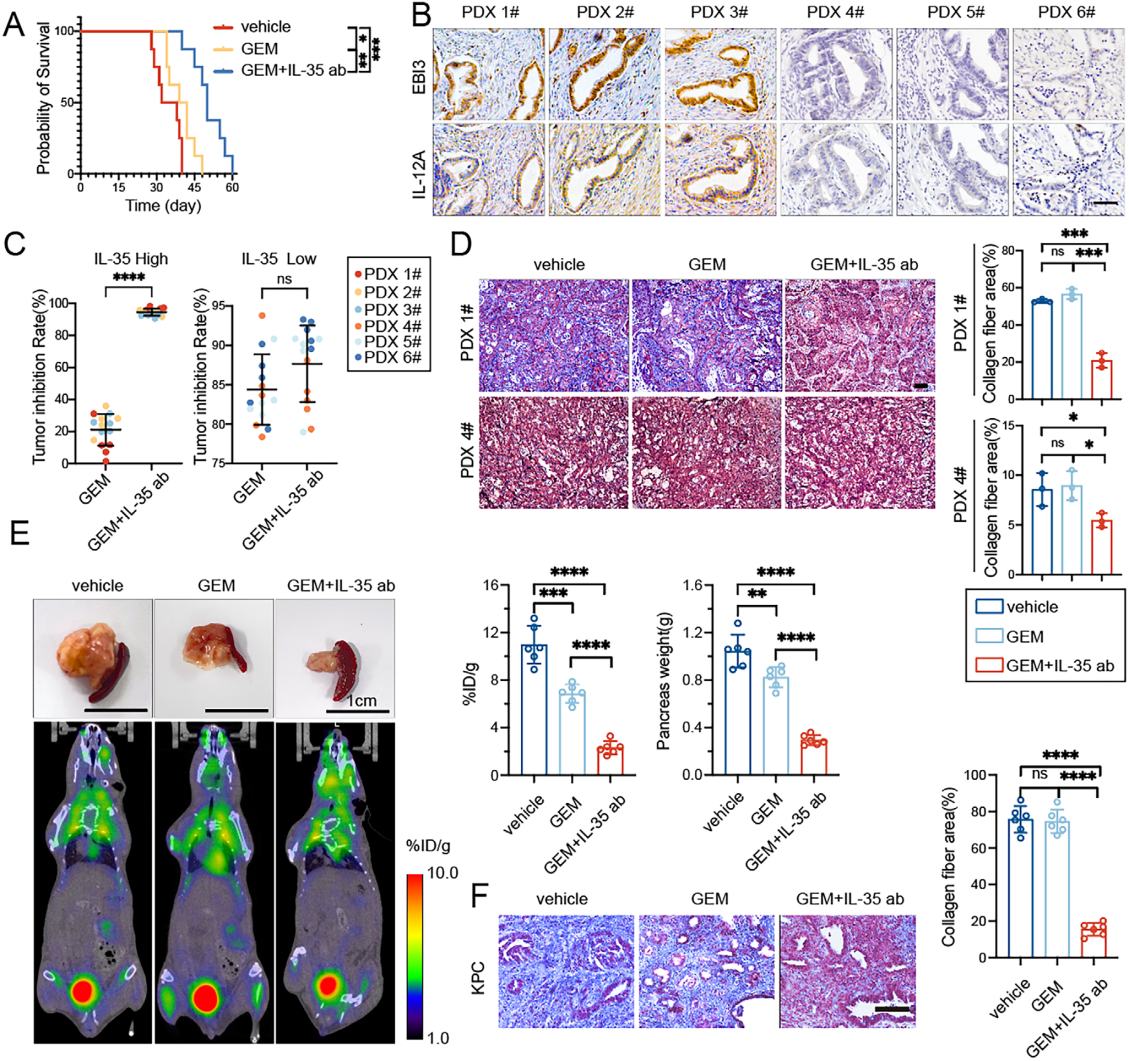
Anti‐IL‐35 efficiently diminishes fibrosis in PDAC while improving the effectiveness of gemcitabine treatment. A) KPC cell lines were implanted into the pancreas of 5‐week‐old C57BL/6 mice to form tumors. The mice were then treated with either saline vehicle, Gemcitabine alone (15 mg kg^−1^), or Gemcitabine in combination with anti‐IL‐35 (25 µg) twice a week. Kaplan‐Meier analysis comparing the survival of indicated group (n =8). B) Representative images of IHC staining of tumor sections from patients with 3 cases of high‐expression (PDX 1#, PDX 2#, PDX 3#) and low‐expression (PDX 4#, PDX 5#, PDX 6#) of IL‐35 in PDX models.C) Tumor inhibition rate of various treatment groups in PDX tumor compared to the control group. D) Sectioning of the indicated PDX tumor after embedding in paraffin was performed for Masson trichrome staining to determine the degree of tissue fibrosis. Representative images (left) and collagen fiber percentage (right) were shown. E) KPC mice were administered different treatment regimens, including saline vehicle, GEM alone (15 mg kg^−1^), or a combination of GEM and anti‐IL‐35 (25 µg) twice a week. The effects of these treatments on pancreatic tumors in KPC mice were evaluated using macroscopic imaging and tumor burden measurement using PET‐CT scans after 14 days of treatment. Scale. Representative images of these assessments were provided. F) Sectioning of the indicated KPC pancreatic tumor after embedding in paraffin was performed for Masson trichrome staining to determine the degree of tissue fibrosis. Representative images (left) and collagen fiber percentage (right) were shown. Data were analyzed using unpaired t‐test. Shown are mean ±SD; *p <.05. **p <.01. *** *p* <.001. ****p <.0001. ns no significance. Scale bars, 100 µm.

Given the crucial role of tumor‐derived IL‐35 in activating PSCs and mediating PDAC fibrosis, which in turn promotes the proliferation, invasion, immunosuppression, and drug resistance of PDAC cells, targeting IL‐35 may be a promising strategy to reduce fibrosis and enhance the effectiveness of GEM‐based chemotherapy. To test this hypothesis, we established pancreatic orthotopic tumor models using KPC‐derived cell lines in C57BL/6 mice, and treated them with the vehicle, GEM alone, or a combination of GEM and anti‐IL‐35 (anti‐EBI3). Compared to the control group, GEM monotherapy moderately improved the survival of tumor‐bearing mice, whereas the combination of GEM and anti‐IL‐35 treatment significantly prolonged survival. We recorded a median survival time of 35 days in control mice, 40.5 days in the GEM monotherapy group, and 50 days in the GEM and anti‐IL‐35 combination therapy group (**Figure** **7**A).

Next, we selected six patient‐derived xenograft (PDX) models in our laboratory, three with high IL‐35 expression and three with low IL‐35 expression (Figure 7B), and subjected them to vehicle control, GEM, and a combination of GEM and anti‐IL‐35 treatments. PDX tumors with high IL‐35 expression exhibited resistance to GEM chemotherapy, with a modest average tumor inhibition rate of only 21.09%. However, the anti‐IL‐35 antibody significantly augmented the efficacy of GEM chemotherapy, increasing the tumor inhibition rate to 94.42%. In contrast, tumors expressing low levels of IL‐35 were highly responsive to GEM chemotherapy, showing an average tumor inhibition rate of 84.38%, which was comparable to that of the group treated with a combination of anti‐IL‐35 and GEM (87.67%) (Figure 7C; Figure S12A,B, Supporting Information). Furthermore, Masson's trichrome staining results indicated that, compared to the low IL‐35 expression group, tumor tissues with high IL‐35 expression had increased PSC activation and fibrosis levels, which were effectively mitigated by the anti‐IL35 antibody, but not by GEM (Figure 7D; Figure S12C, Supporting Information).

These findings were further supported by the results obtained from the KPC spontaneous tumor model. Although GEM monotherapy moderately reduced the tumor burden in KPC mice, it failed to inhibit PSC activation or decrease tumor tissue fibrosis. Conversely, the combination with anti‐IL‐35 showed a highly favorable antitumor effect, significantly suppressing tumor growth and reducing both PSC activation and tissue fibrosis levels (Figure 7E,F; Figure S12D, Supporting Information).

Given the increasing clinical adoption of combination regimens, we also evaluated the effect of anti‐IL‐35 with the GEM plus nab‐paclitaxel (A+G) regimen. In IL‐35 high PDX models, anti‐IL‐35 combined with A+G led to a marked suppression of tumor progression, and in KPC spontaneous tumor models, this combination produced the strongest inhibition of tumor growth, PSC activation, and fibrosis compared with A+G alone (Figure S13, Supporting Information).

Taken together, these data demonstrate that IL‐35 blockade has limited efficacy as a monotherapy but significantly potentiates the antitumor effects of GEM‐based regimens, especially in IL‐35 high PDAC. Therefore targeting IL‐35 represents a promising strategy for reducing desmoplasia and improving the therapeutic response to first‐line chemotherapy.

## 3 Discussion

PDAC is a highly invasive and malignant tumor known for its high rates of treatment resistance and recurrence,^[^ ^31^, ^32^ ^]^ primarily due to the presence of severe fibrosis in the TME.^[^ ^33^ ^]^ aPSCs play a crucial role in pancreatic cancer fibrosis, contributing to the malignant behaviors of PDAC.^[^ ^2^, ^23^, ^34^ ^]^ However, the mechanisms underlying the interaction between tumor cells and PSCs that lead to PSC activation remain unclear. In this study, we demonstrated that tumor‐derived IL‐35 is essential for PSC activation and subsequent induction of fibrosis. Furthermore, targeting IL‐35 effectively inhibited PSC activation and reduced fibrosis, thereby enhancing the efficacy of GEM‐based chemotherapy. Thus, this study revealed new roles and mechanisms of IL‐35 in the malignant progression of PDAC, expanded our understanding of the bioactivity of IL‐35 under normal and pathological conditions, and provided new potential strategies for the clinical treatment of PDAC.

The receptors for IL‐35 are composed of IL‐12Rβ2 and GP130. These receptors can assemble into heterodimeric complexes containing both IL‐12Rβ2 and GP130, or form homodimeric complexes containing either IL‐12Rβ2 and GP130. These different receptor complexes induce diverse cellular responses to IL‐35 stimulation.^[^ ^33^ ^]^ Consistent with these findings, our data demonstrated that IL‐35 binds to IL12Rβ2:GP130 heterodimer to induce IGFBP2 expression, and to the GP130:GP130 homodimer to enhance Tsp‐1 expression, in PDAC cells (Figure 5F). When IL‐35 binds to its receptor, intracellular signaling events are initiated, leading to the activation of the downstream transcription factors STAT1 and STAT4. These activated transcription factors form homodimers or heterodimers that subsequently regulate the expression of genes involved in immune responses and inflammation.^[^ ^35^ ^]^ ChIP‐reChIP analysis revealed that IL‐35 induces the formation of the pSTAT1:pSTAT4 heterodimer, which binds to the *IGFBP2* promoter, and promotes the formation of the pSTAT1:pSTAT1 homodimer, which binds to the *THBS1* promoter (Figure 5G). In addition, dual‐luciferase assays indicated that these binding sites were required for IL‐35‐induced transcriptional activation of *IGFBP2* and *THBS1* (Figure 5F). Taken together, these results suggest that STAT1:STAT4 heterodimers and STAT1:STAT1 homodimers are likely to directly activate the transcription of IGFBP2 and THBS1, respectively, by binding to their promoter regions. We found that the STAT1:STAT4 heterodimer and STAT1:STAT1 homodimer bind to different sites on the *IGFBP2* and *THBS1* promoters, although all these sites are predicted to contain the STAT consensus binding sequences. It is highly likely that the binding specificity of STAT proteins to these sites is governed by sequences surrounding the STAT consensus.

In this study, we demonstrated that tumor‐derived IL‐35 is crucial for PSC activation, but does not directly activate PSCs. Further results indicate that IGFBP2 and Tsp‐1 are downstream effectors of IL‐35, which is involved in PSC activation. IGFBP2, which is primarily recognized as an IGF‐binding protein, contributes to the regulation of IGF bioavailability and activity.^[^ ^36^, ^37^ ^]^ When bound to IGFs, particularly IGF‐1, IGFBP2 modulates their distribution, stability, and interactions with IGF‐1R. This interaction subsequently influences the activation of downstream signaling pathways such as PI3K‐Akt and MAPK, which are implicated in cellular processes such as cell proliferation, survival, migration, and differentiation.^[^ ^31^ ^]^ Notably, our data revealed that IGFBP2 promotes the activation of the IGF‐1R‐PI3K‐Akt signaling cascades, leading to PSC activation and fibrosis. Tsp‐1, an ECM glycoprotein, is a major activator of latent TGF‐β1, a key mediator of fibrosis.^[^ ^30^, ^38^, ^39^ ^]^ Tsp‐1 binds to latent TGF‐β, converting it into its biologically active form, which then engages with cell surface receptors. Activated TGF‐β receptors phosphorylate Smad2 and Smad3, leading to transcriptional activation of target genes associated with fibrosis and various cellular processes.^[^ ^38^ ^]^ Consistently, treatment with LSKL, a competitive TGF‐β1 antagonist, effectively blocked Tsp‐1‐induced PSC activation, indicating that Tsp‐1 activates the TGF‐β signaling cascade, inducing PSC activation and fibrosis. The detailed molecular mechanisms by which TGF‐β contributes to PSC activation are still unknown, but they potentially involve intricate crosstalk with other signaling pathways; additional research is needed to elucidate these complexities. Furthermore, we demonstrated that inhibiting either the IGFBP2 or Tsp‐1 signaling pathway alone was not sufficient to completely block IL‐35‐induced PSC activation, suggesting that these two pathways are independent. However, the relative contributions of these two pathways to IL‐35‐induced PSC activation remain unclear and require further investigation.

Allograft tumor, PDX, and KPC spontaneous tumor models have provided compelling evidence for the promising therapeutic potential of anti‐IL‐35 in mitigating PDAC fibrosis and improving the efficacy of GEM‐based chemotherapy. IL‐35, an immunosuppressive IL‐12 family heterodimeric cytokine composed of IL‐12A and the EBI3 subunits, shares a subunit with both IL‐12 and IL‐27.^[^ ^40^, ^41^ ^]^ In this study, we used a neutralizing antibody against EBI3 to block IL‐35 activity, which is a commonly accepted method.^[^ ^22^ ^]^ However, this approach may interfere with IL‐27 signaling, potentially causing side effects.^[^ ^42^ ^]^ IL‐27 is known to promote T cell activation and interferon‐γ production while also regulating inflammatory responses; thus, its inhibition may theoretically impair anti‐tumor immunity or enhance systemic inflammation. Nevertheless, we did not specifically measure IL‐27–related cytokines or downstream signaling activity, which we recognize as a limitation. Future work will include a comprehensive safety assessment, such as cytokine profiling and histopathological analysis, to fully evaluate the effect of IL‐35 blockade on IL‐27 signaling and immune homeostasis. An ideal strategy would involve the development of bispecific antibodies targeting both IL‐35 subunits simultaneously, avoiding interference with the IL‐12 and IL‐27 signaling pathways. Unfortunately, owing to the considerable challenges in developing bispecific antibodies targeting IL‐35, there are currently no reported successes. However, this approach remains crucial for the future development of IL‐35 targeting strategies. Our previous study indicated that IL‐35 plays a role in pancreatic cancer angiogenesis through a vascular endothelial growth factor‐independent mechanism.^[^ ^22^ ^]^ Additionally, IL‐35 promoted GEM resistance in PDAC cells by eliminating reactive oxygen species (ROS) accumulation.^[^ ^43^ ^]^ Numerous studies have highlighted its critical role in shaping the immunosuppressive TME, which could potentially contribute to fibrosis.^[^ ^44^ ^]^ Therefore, the improved fibrosis reduction and enhanced efficacy of GEM chemotherapy through IL‐35 inhibition may not only result from the suppression of PSC activation but also from the inhibition of angiogenesis, remodeling of the immunosuppressive microenvironment, and elimination of ROS.

Our study has several limitations. First, although our data indicate that IL‐35 is preferentially linked to the myCAF phenotype, its subtype‐specific effects on human PSCs remain unclear. Future studies integrating single‐cell and spatial transcriptomic approaches are needed to fully elucidate these interactions and their contributions to stromal remodeling. Second, while IL‐35‐mediated PSC activation was consistently observed in both non‐SCID and immunocompetent C57BL/6J models, suggesting that this process is independent of the presence of immune cells, the broader immunological impact of IL‐35 has not been comprehensively profiled. Our supplementary flow cytometry data indicated that IL‐35 shapes an immunosuppressive niche, including the expansion of Foxp3⁺ Tregs and M1‐like tumor‐associated macrophages (Figure S14, Supporting Information). Given the substantial divergence between murine and human immune systems, future studies should incorporate humanized mouse models and deep immune profiling to enhance translational relevance. Third, although the sample size of our in vivo cohorts (n = 6–8 per group) was comparable to that of many PDAC studies, no formal power calculation was performed; thus, these findings should be interpreted as exploratory and validated in larger, independent cohorts. Finally, transcriptomic profiling revealed that IL‐35 activated MAPK signaling and cytokine–cytokine receptor interaction pathways in PDAC cells (Figure S15, Supporting Information), implicating IL‐35 in broader protumorigenic programs beyond stromal activation. These insights provide a strong rationale for therapeutic strategies combining IL‐35 blockade with MAPK inhibitors or other immunoregulatory approaches (e.g., IL‐6/STAT3 or CXCL pathway inhibition) with the potential to reprogram the TME and improve the efficacy of standard treatments.

In conclusion, this study has unveiled a new function of IL‐35 in PSC activation and fibrosis and elucidated the underlying mechanisms. We identified the combination of anti‐IL‐35 and GEM‐based chemotherapy as a promising strategy for PDAC treatment. These findings expand our understanding of the role of IL‐35 in the malignant progression of PDAC, and offer new potential strategies for clinical treatment.

## 4 Experimental Section

### Human Sample Collection

A total of 109 tissue samples were retrospectively collected from patients with PDAC who underwent curative R0 resection at the Tianjin Medical University Cancer Institute and Hospital between July 2011 and January 2015. The follow‐up rate of these patients was 100%. Additionally, 12 fresh PDAC tissue samples of PDAC were collected from January 2020 to December 2022. The use of these specimens and patient information was approved by the Ethics Committee of the Tianjin Medical University Cancer Institute and Hospital. Written informed consent was obtained from all patients for the use of their specimens and disease information in future investigations, in accordance with the Ethics Committee guidelines and the ethical principles of the Declaration of Helsinki.

### Isolation of Mouse Primary PSCs

The mouse pancreases were removed and cut into small pieces. A preprepared pancreatic enzyme solution, containing 0.1% trypsin (Sigma, T2600000) and 0.05% collagenase IV (1 mg mL^−1^, Sigma, C4‐BIOC) in GBSS (Sigma, G9779) supplemented with 0.1% bovine serum albumin was added. The tissues were digested in a constant temperature shaking incubator at 37 °C for 15 min. Primary PSCs were obtained by density gradient centrifugation and cultured in Dulbecco's modified DMEM medium (DMEM) for subsequent experiments.

### Isolation of Human Primary PSCs

To ensure cell viability, it is crucial to collect samples from surgically resected pancreatic tissues immediately after surgery. Pancreatic tissue samples were cut into small fragments of approximately 1–2 mm^3^. The cells were then dispersed in Dispase II (2 mg mL^−1^, Sigma, 42613‐33‐2), Collagenase IV (1 mg mL^−1^, Sigma, C4‐BIOC), and DNase I (1 unit/mL, NEB, M0303S). PSCs were separated by density gradient centrifugation. Finally, the purified PSCs were cultured in DMEM medium with 10% fetal bovine serum for subsequent experiments.

### Mouse Models of Tumors

The Tianjin Medical University Cancer Institute and Hospital Ethics Committee approved all animal experiments, which were conducted by skilled investigators under an approved protocol. Female SCID or C57BL/6 mouse models (5‐weeks‐old) were used. All the mice were maintained under specific pathogen‐free conditions. Tumor cells (1 × 10^5^) were orthotopically injected to induce tumor development. After 5 or 6 weeks, the tumors were harvested from the mice in each group.

LSL‐Kras^G12D/+^, LSL‐Trp53^R172H/+^ and Pdx1‐Cre (KPC) mouse models were generated in‐house and genotyped as previously described.^[^ ^45^ ^]^ Two‐month‐old KPC mice with tumors of similar sizes in situ were randomized into three groups and treated with various treatment options. Vehicle was used as a negative control, GEM was administered twice a week at a dose of 15 mg kg^−1^ per mouse, and anti‐IL‐35 (anti‐EBI3) was administered twice a week at a dose of 25 µg per mouse.

IL‐12A^flox/flox^ and EBI3^flox/flox^ mice were purchased from the Shanghai Model Organisms Center, PR China.

Group sizes (n = 6–8) were chosen according to common practice in PDAC mouse studies^[^ ^46^, ^47^, ^48^ ^]^ and in compliance with the “3R” (Replacement, Reduction, Refinement) principles. Sample sizes were guided by pilot data and observed effect sizes,^[^ ^21^, ^22^, ^43^ ^]^ while balancing feasibility, given the multiple time points and extensive analyses. Formal a priori power calculations were not performed, and the results should be considered exploratory.

### Oil Red O Staining

PSCs were fixed with 4% paraformaldehyde for 10 min, washed with phosphate‐buffered saline, and incubated with Oil Red O staining solution (Sigma–Aldrich, O9755) for 30 min. After washing with deionized water, the cells were analyzed, and images of the stained cells were captured using a Zeiss microscope for imaging and analysis. Oil Red staining was quantified by calculating the proportion of Oil Red–positive lipid droplet–retaining cells relative to the total number of cells in the field. Specifically, cells with abundant lipid droplets were considered quiescent, whereas those with a loss of lipid droplets were considered activated. At least five randomly selected fields per sample were analyzed, and the activation ratio was expressed as the percentage of activated cells over the total fibroblast population.

### Masson's Trichrome and IHC Staining

For Masson's trichrome staining, the tissue sections were prepared using a modified Masson's trichrome stain kit (Cat. No. G1346, Solarbio, Beijing, China). Images were acquired using a KENYENCE microscope, and the collagen fiber area was analyzed using ImageJ software by calculating the percentage of collagen stained in blue within the field of view.

IHC staining was used to detect IL‐35, α‐SMA, COL‐1, IGFBP2, and Tsp‐1 expression in pancreatic tumor tissues. Briefly, paraffin‐embedded sections of human PDAC or mouse tumors were deparaffinized and heated to retrieve the antigens. The sections were incubated with primary antibodies overnight at 4 °C, followed by incubation with an HRP‐conjugated secondary antibody at 37 °C for 30 min. A DAB Substrate Kit (ZLI‐9028, ZSGB‐Bio, China) was used to perform the chromogenic reactions. The staining intensity was evaluated using the following criteria: 0, negative; 1, low; 2, medium; and 3, high. The extent of staining was scored as follows: 0, 0% stained; 1, 1–25% stained; 2, 26–50% stained; and 3, 51–100% stained. Five random fields (×100 magnification) were evaluated under a light microscope. The final scores were calculated by multiplying the intensity scores with the extent of the scores. Samples were divided into four grades: 0, negative (−); 1–2, low staining (+); 3–5, medium staining (++); and 6–9, high staining (+++).

Multiplex fluorescent IHC assays were performed according to the manufacturer's instructions (Perkin Elmer, 2395285) for immunological assessment of α‐SMA, COL‐1, and cytokeratin 19.

### PDX Establishment

In the present study, PDX models were used for cancer research. Initially, fresh tumor samples were obtained from primary cancers. These tumor tissues were subsequently transplanted into immunodeficient mice. Five‐week‐old NSG mice, under pathogen‐free conditions, were used for patient‐derived xenograft transplantation. Briefly, a small incision was made subcutaneously and primary PDAC samples were minced into 1 mm^3^ fragments and injected directly into the mouse incision. The incision was closed using sutures. The tumor formation was monitored after implantation. The PDX carried mice from the same case group were randomly divided into five groups. The treatment was performed when the longest diameter of xenografts reached 5 mm, GEM (15 mg kg^−1^, twice a week) and anti‐IL‐35 (25 µg, twice a week) were given to the PDX carrying mice. The tumor size was measured weekly using calipers. After 35 days, the mice were sacrificed, and the tumors were collected and sectioned. Tumor volume was calculated as follows: volume = (length × width × width) /2. Establishing PDX models provides a research platform that more accurately reflects the characteristics of primary tumors. Using this model, further drug efficacy testing and biological analyses will be conducted to gain deeper insights into the biological behavior of tumors and their responses to medications.

### Statistical Analysis

Statistical analyses were performed using IBM SPSS Statistics 26 and Prism 9.0 software. Each experiment was conducted in triplicate, and data are presented as mean ± standard deviation. Spearman's correlation analysis was used to assess these relationships. Unpaired t‐tests were used to compare mean values. Kaplan–Meier curves were used to analyze the median survival. Statistical significance was set at p < 0.05. One‐way analysis of variance was followed by Tukey's post‐hoc test for multiple group comparisons, whereas two‐tailed unpaired t‐tests were used for pairwise comparisons. Additional information is available in the Supporting Information.

This work was supported by the National Natural Science Foundation of China (grants 82272680, 82072659, 82030092, 82272799, 82072657); National Key Research and Development Program of China(2021YFA1201100); Tianjin Key Medical Discipline (Specialty) Construction Project (TJYXZDXK‐009A); Tianjin Municipal Education Commission Research Program Project (2024KJ190); Natural Science Foundation of Tianjin (24JCQNJC00710).

## Conflict of Interest

The authors declare no conflict of interest.

## Author Contributions

H.L., H.S., J.L., and Y.W. contributed equally to this work. C.H., J.H., A.C., and Y.F. conceived and designed the whole research; H.L., H.S., and J.L. performed most of the assays and data analyses; L.W., J.L., Y.Y., and P.X. performed some experiments; C.X., G.L., and Y.G. provided technical support. A.C., C.H., H.L., H.S., and Y.W. wrote, reviewed, and revised the manuscript.

## Supporting information



Supporting Information

Supporting Information

Supporting Information

## Data Availability

The data that support the findings of this study are available in the supplementary material of this article.
