# Supplementary material for: Tumor‐Derived Interleukin 35 Promotes Fibrosis in the Tumor Microenvironment of Pancreatic Cancer by Activating Pancreatic Stellate Cells
Source: Adv Sci (Weinh). 2025 Nov 14;13(5):e09074. doi: 10.1002/advs.202509074 (PMC12850332; doi:10.1002/advs.202509074)
Supplement: Supplementary file 1 — Supporting Information [file ADVS-13-e09074-s001.docx]

Supporting Information

Tumor-derived Interleukin 35 Promotes Fibrosis in the Tumor Microenvironment of Pancreatic Cancer by Activating Pancreatic Stellate Cells

*Authors: Hui Li, Huizhi Sun, Jing Liu, Yan Wu, Lin Wei, Jianming Li, Yudong Yuan, Peng Xie, Chao Xu, Guolu Luo, Yuqi Guan, Yukuan Feng^*^, Antao Chang^*^, Jihui Hao^*^, Chongbiao Huang^*^*

**Supplementary Materials and Methods**

***Cell culture:*** Human PDAC cell lines BxPC-3, PANC-1, CFPAC-1, and MIA PaCa2 were acquired from the American Type Culture Collection (ATCC). The murine cell line from the KPC (LSL-Kras^G12D/+^; LSL-Trp53^R172H/+^; Pdx-1-Cre) mouse was generously provided by Dr. Tingbo Liang at the Department of Surgery of the Second Affiliated Hospital, Zhejiang University, China. These cells were cultured at 37°C in a humidified atmosphere of 95% air and 5% CO2 with DMEM, RPMI-1640 and IMDM basic medium supplemented with 10% fetal bovine serum (FBS).

***Immunofluorescence staining:*** Immunofluorescence staining was performed on PSC cells. Briefly, PSCs were treated with conditioned medium and then incubated with anti-α-SMA (1:400) or anti-COL-1 (1:200) antibodies at 4 °C overnight. The cells were then incubated with fluorescent dye-labelled secondary antibodies at room temperature for 2 h. Following this, the cells were incubated with an anti-fade 4,6-diamidino-2-phenylindole (DAPI) solution (1:1,000). Images were captured with a confocal fluorescence microscope, and the positive area was analyzed using Image J software.

***Collagen contraction assay:*** A total of 1x10^4^ PSCs were mixed with 150 μL of mouse tail type 1 collagen (Corning, 354236), neutralized with sodium hydroxide, and seeded in 24-well plates. The PSC/collagen mixture was allowed to solidify for 30 minutes before the corresponding treatments were added to each well. After 7 days, the gels were imaged under identical conditions. The gel contraction was quantified using ImageJ (NIH, Bethesda, MD) by measuring the remaining gel area. The contraction index was calculated as (1 - contracted area / original area), thereby representing the proportion of area reduction relative to the initial gel area. Each experiment was independently repeated three times, and the mean contraction index ± SD was reported.

***Human XL Cytokine Array:*** Supernatants (500 µL) from PANC-1 cells, with or without IL-35 up-regulation, were analyzed using the Proteome Profiler Human XL Cytokine Array Kit (ARY022B, R&D systems) following the manufacturer's instructions. Data shown were from a 3-minute exposure to X-ray film. Profiles of mean spot pixel density were created using image J software analysis.

***Reverse transcription PCR:*** Total RNA was extracted using TRIzol reagent (Invitrogen) and converted to cDNA through reverse transcription (Bimake). Real-time fluorescent qPCR was then conducted, with each RT-PCR experiment independently repeated at least three times. ACTB was used as the loading control.

***EdU cell proliferation assay:*** 1x10^4^ cells were seeded into a 96-well plate and subjected to different treatments. EdU reagent was added to the culture medium to a final concentration of 10μM. The cells were cultured in an incubator for 2 hours, then fixed with 4% formaldehyde for 10 minutes. Edu staining was performed using the Edu staining kit (Abcam, ab222421) to label Edu-positive cells with fluorescence. Fluorescence labeling was observed and imaged using a fluorescence microscope. The number of Edu-positive cells was analyzed using Image J software. Each experiment was independently repeated three times.

***Transwell Assay:*** Transwell assays were conducted using 24-well plates with 8.0 μm pore inserts. BXPC-3 or PANC-1 cells (5 × 10^4^ cells/mL) were seeded in 200 μL into the upper chamber. The basolateral chambers were filled with 600 μL of conditioned medium with specific compositions for different assays. For chemotaxis assays, the upper chamber contained FBS-free medium, and the basolateral chamber contained 50% total medium + 50% supernatants from the indicated cells. For migration assays, the upper chamber contained 50% FBS-free medium + 50% supernatants, and the basolateral chamber contained 50% total medium + 50% supernatants. For invasion assays, the upper chamber contained FBS-free medium and 50 μL Matrigel, and the basolateral chamber contained 50% total medium + 50% supernatants. The incubation lasted 6 to 12 hours. Migrated cells in 5 random fields at 200× magnification was counted for each insert. Each experiment was performed in triplicate, and mean values are presented.

***Real-Time CelI AnaIysis (RTCA):*** Real-time cell analysis was performed using the xCELLigence RTCA DP system (ACEA Biosciences). The cell index (CI) is a dimensionless parameter automatically calculated by the instrument, reflecting changes in electrical impedance across microelectrodes at the bottom of each well. CI values are proportional to the number, morphology, and adhesion status of cells, thereby serving as a quantitative readout of cell growth and viability in real time. Grow 2000 cells based on the experimental requirements. Ensure cells are in the logarithmic growth phase and prepare a cell suspension. Turn on the RTCA system and launch the associated software. Before seeding the cells, add 100 μL culture medium to each well of the RTCA plate. Place the RTCA plate in the analyzer to perform background calibration. After background calibration, remove the culture medium from the RTCA plate. Add 100 μL CM to each well. Place the RTCA plate back into the analyzer and record the initial cell index (CI) values. After the experiment, export the cell index data recorded by the RTCA system. Use the RTCA software analysis tools to analyze changes in the cell index.

***Plasmid construction and stable cell line establishment:*** The human or mouse fused EBI3-IL-12A genes were amplified by PCR using commercial IL-35-overexpressing plasmids (InvivoGen) as templates. The fused IL-35 genes were then cloned into pLV-EF1-MCS-IRES-Bsd vectors (Biosettia) for stable expression. Lentiviruses were produced in 293T cells following the manufacturer’s instructions. An empty vector was transfected into control cells. Tumor cells (1 × 10^5^ cells in 2 mL medium with 0.8 μg/mL polybrene) were infected with 1 mL of lentivirus supernatant containing IL-35 genes. Empty vector-transfected cells served as controls. Forty-eight hours post-infection, blasticidin (InvivoGen) was added to the culture medium to select for cells stably expressing IL-35 genes.

For the cell lines with stable knockdown, shRNA sequences targeting *IL-12A*, *EBI3*, *IGFBP2*, and *THBS1* genes were designed using Biosettia’s shRNA designer (http://biosettia.com/support/shrna-designer). The designed shRNA sequences were synthesized and cloned into pLV-hU6-EF1α-puro or pLV-mU6-EF1α-puro vectors (Biosettia) for stable knockdown. Lentiviruses were then produced in 293T cells. Scrambled sequences were transfected into control cells. Tumor cells were simultaneously infected with lentiviruses carrying shIL-12A and shEBI3 sequences to knock down IL-35 expression, infected with lentiviruses carrying shIGFBP2 or shTHBS1 sequences to knock down IGFBP2 or THBS1 expression. Cells infected with scrambled sequences served as controls. The most efficient stable cell lines generated through these processes were selected for subsequent experimental assays.

***Dual Luciferase assay:*** The Dual-Luciferase Reporter Assay Kit (Promega, E2920) was used according to the manufacturer’s instructions. In brief, 293T cells were transfected with the specified luciferase reporter plasmids. After 48 hours, cells were washed with PBS and lysed using 1× Passive Lysis Buffer (PLB) for 30 minutes at room temperature. Following cell lysis, an aliquot of the lysate was used to measure luciferase activity. First, the luminescent substrate for firefly luciferase was added, and luminescence (RLU1) was measured using a luminometer. Next, 1× Stop & Glo® Reagent was added to quench the firefly luciferase reaction and simultaneously initiate the Renilla luciferase reaction. Renilla luciferase activity (RLU2) was then measured. The results were expressed as fold induction relative to control cells after normalization to Renilla luciferase activity to correct for differences in transfection efficiency and cell number. Each experiment was independently performed three times to ensure reproducibility and reliability of the results.

***ChIP and ChIP: reChIP Assays:*** ChIP experiments were conducted using the ChIP assay kit (Millipore, 17-371) following the manufacturer's guidelines. Briefly. PANC-1 cells were pre-treated with IL-35 (100 ng/mL) or left untreated, followed by immunoprecipitation using either anti-STAT1 (CST, 9172, 10μg/100μL) or anti-STAT4 (CST, 2653, 10μg/100μL) antibodies. Immunoprecipitated products were analyzed by RT-PCR. For the repeat ChIP analysis of the ChIP experiments, immunoprecipitation was initially performed using anti-STAT1 antibody, followed by a second round of immunoprecipitation using anti-STAT4 (S1→S4).

***Western blot:*** Whole-cell extracts were prepared by lysing the cells with SDS protein lysis buffer supplemented with proteinase inhibitor cocktail (Roche). The protein lysates were then separated by SDS-PAGE. Following electrophoresis, the proteins were transferred to PVDF membranes. The membranes were blocked with 5% non-fat dry milk in TBST (Tris-buffered saline with 0.1% Tween-20) for 1 hour at room temperature. After blocking, the membranes were incubated overnight at 4°C with primary antibodies specific to the target proteins. Following primary antibody incubation, the membranes were washed and then incubated with secondary antibodies (goat anti-rabbit or goat anti-mouse, CST) diluted 1:5000 in blocking buffer for 1 hour at room temperature. The targeted proteins were detected using an enhanced chemiluminescence (ECL) detection system, and the resulting bands were visualized using an appropriate imaging system.

***Single-cell RNA-seq Data Analysis:*** Publicly available single-cell RNA sequencing (scRNA-seq) data of human PDAC tissues (GSA: CRA001160) were downloaded and processed using the Seurat (v4.3.0) R package. All functions were run with default parameters, unless specified otherwise. t-distributed stochastic neighbor embedding (t-SNE) was applied for dimensionality reduction and visualization of cell populations. Major cell types were annotated based on canonical marker genes. Cancer-associated fibroblast (CAF) subclusters were further classified into myCAF, iCAF, and apCAF populations based on published gene signatures. The relative abundance of each CAF subtype was calculated as the proportion of cells in each subcluster normalized to the total CAF population per sample. To quantify IL-35 expression, an integrated IL-35 score was calculated by averaging the normalized expression values of IL12A and EBI3 at the single-cell level, followed by min–max scaling. Samples were then stratified into high- and low-IL-35 groups considering both EBI3 and IL12A expression levels. Five samples with the high IL-35 scores and five with the low scores were selected for comparative analysis. The detailed IL-35 scores, expression levels of EBI3 and IL12A, and the relative proportions of each CAF subtype for all samples are provided in **Supplementary Table 2.** The distribution of CAF subtypes across IL-35 expression groups was compared using two-sided Wilcoxon rank-sum tests. For multiple comparisons across cell clusters or gene sets, P values were adjusted using the Benjamini–Hochberg false discovery rate (FDR) correction, and an adjusted q value < 0.05 was considered statistically significant.

**Supplementary Figures and Figure legends**

**
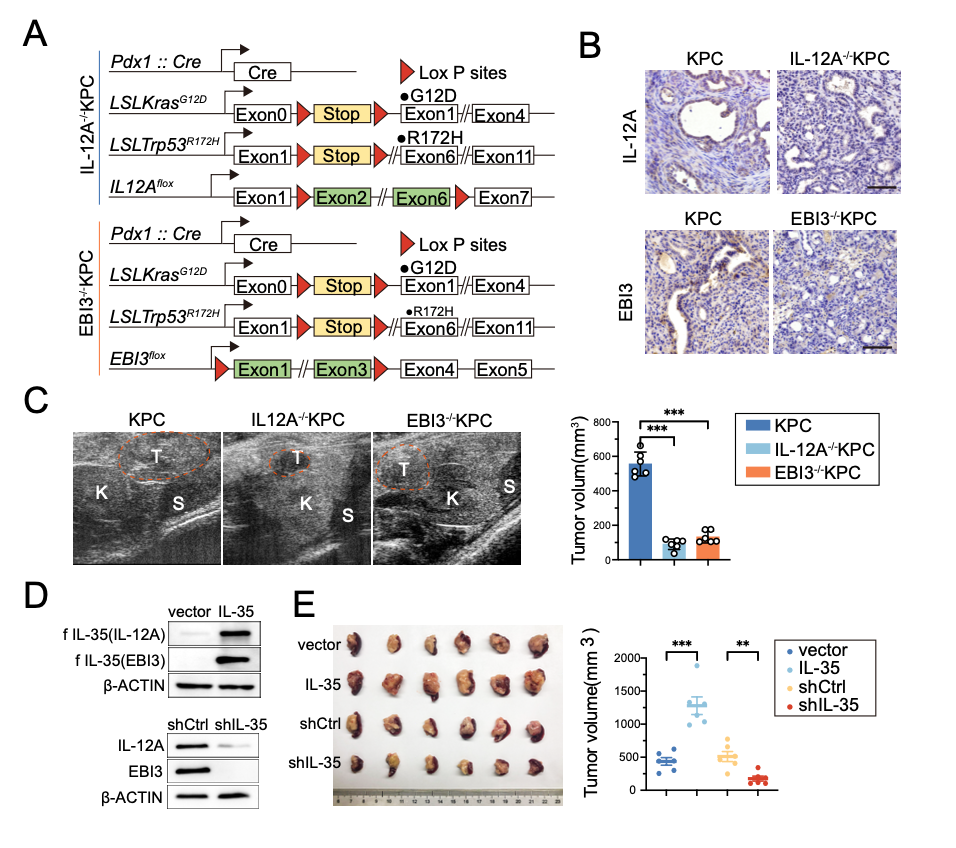
**

**Figure S1.** **Tumor-Derived IL-35 significantly influences tumor growth. A,** Schematics of the EBI3^-/-^KPC and IL12A^-/-^KPC mouse model of PDAC, which uses LSL-Kras^G12D/+^ (K), LSL-Trp53^R172H/+^ (P), Pdx1-Cre (C), pancreas-specific depletion of EBI3 or IL-12A was attained by crossing KPC with the EBI3^flox/flox^ or IL-12A ^flox/flox^ mouse strain and is referred to as EBI3^-/-^KPC or IL12A^-/-^KPC. **B,** Representative images of IHC staining for IL-12A and EBI3 in KPC, IL12A^-/-^KPC and EBI3^-/-^KPC mice. **C,** Tumor size in 3-month-old KPC, IL-12A^-/-^KPC, and EBI3^-/-^KPC mice was measured using ultrasound detection. **D,** The expression level of IL-12A and EBI3 in KPC-vector, KPC-IL-35, KPC-shCtrl and KPC-shIL-35 cell lines. **E,** Orthotopic injection of 1x10^5^ indicated cells into the pancreas of Five-week-old SCID mice (n=6). After 30 days, harvest the tumors. Photos and tumor volume of primary tumors are presented. Data were analyzed using an unpaired t test. Shown are mean ± SD; **p* < .05. ** *p* < .01. *** *p* < .001. Scale bars, 100 μm.

**
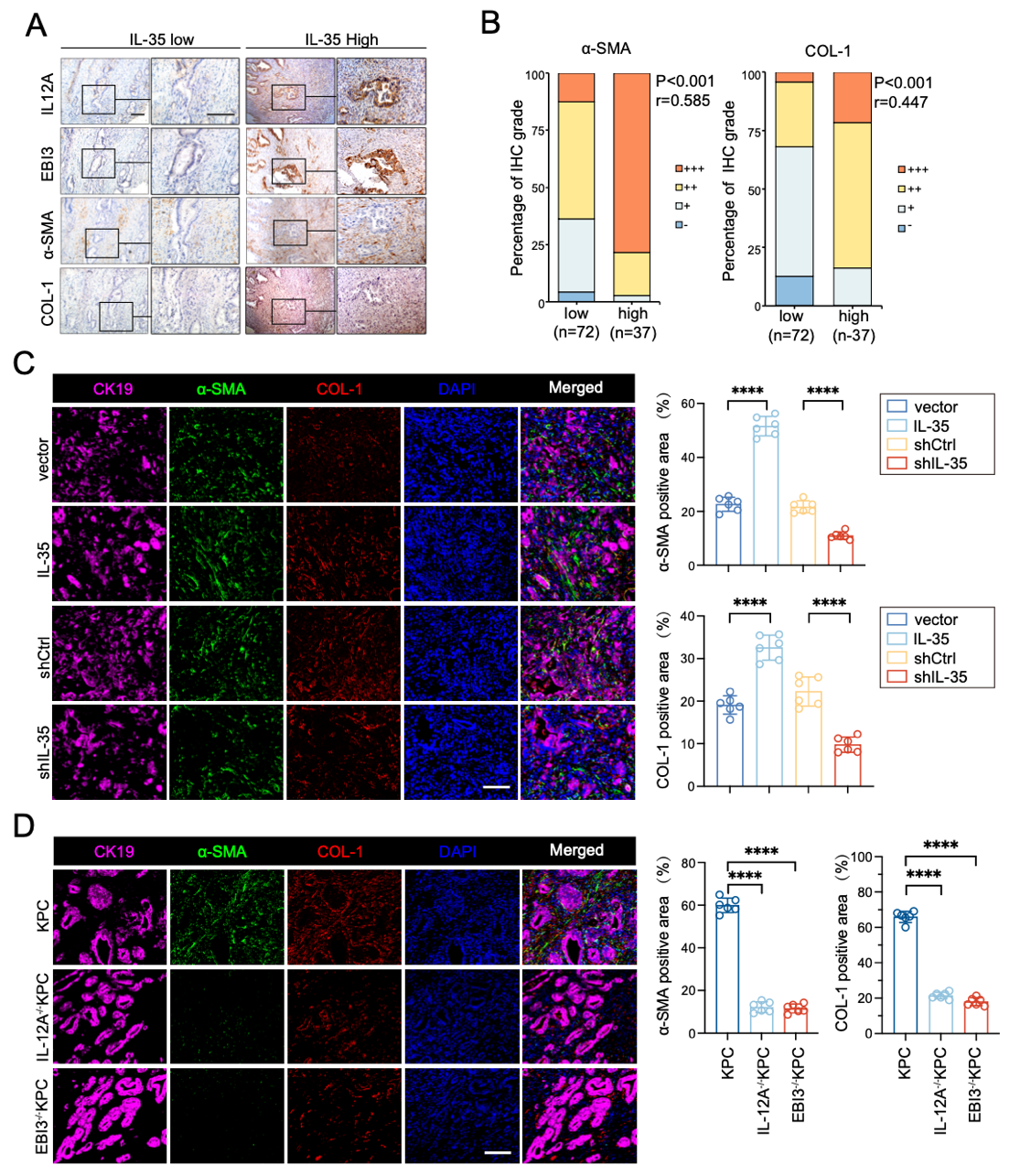
**

**Figure S2. Tumor-Derived IL-35 is integral in pancreatic cancer fibrosis. A,** Representative images of IHC staining for IL-35, α-SMA, and COL-1 (two indicators reflecting tissue fibrosis degree). **B,** Distribution of α-SMA and COL-1 in 109 PDAC slices at different IL-35 expression levels. Data were analyzed using Spearman correlation analysis. **C-D,** Multiplex fluorescent IHC staining of CK19 (cytokeratin 19, an epithelial cell marker), α-SMA, and COL-1 in tumor tissues. **C,** Orthotopic injection of 1x10^5^ indicated cells into the pancreas of Five-week-old SCID mice. Representative images from the indicated group (left) and the positivity of α-SMA/COL-1 (right) are presented. **D,** Representative images from KPC, IL-12A^-/-^KPC, and EBI3^-/-^KPC (tumor diameter was approximately 8 mm) and the positivity of α-SMA/COL-1 (right) are shown. Data were analyzed using an unpaired t test. Shown are mean ± SD; **** *p* < .0001. Scale bars, 100 μm.


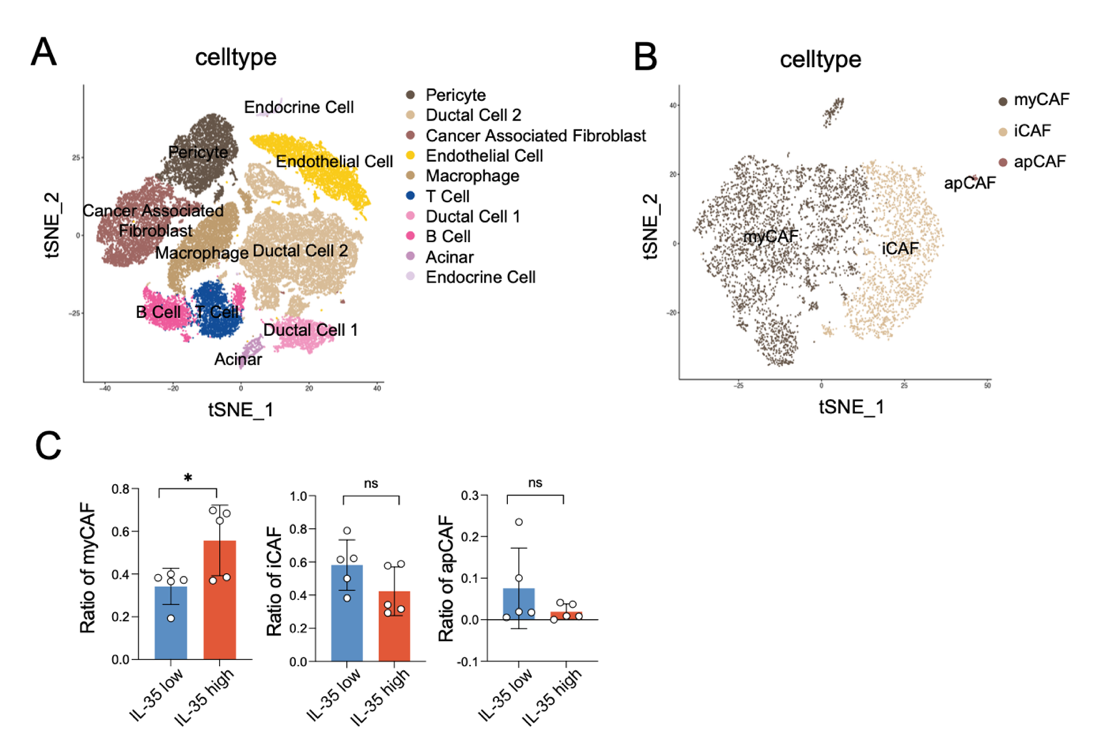


**Figure S3.** **IL-35 preferentially associates with myCAF-like PSC subpopulations. A,** t-SNE visualization of single-cell transcriptomes from PDAC tissues, showing major cell populations annotated by canonical lineage markers **B,** Reclustering of fibroblast populations, revealing distinct CAF subclusters classified as myCAF-like, iCAF-like, and apCAF-like. Each dot represents a single PSC, and colors indicate subcluster identity. **C,** Proportion of CAF subclusters in tumors stratified by IL-35 expression level. Data were analyzed using two-sided Wilcoxon rank-sum test. Shown are mean ± SD, ns. no significance, * *p* < .05.


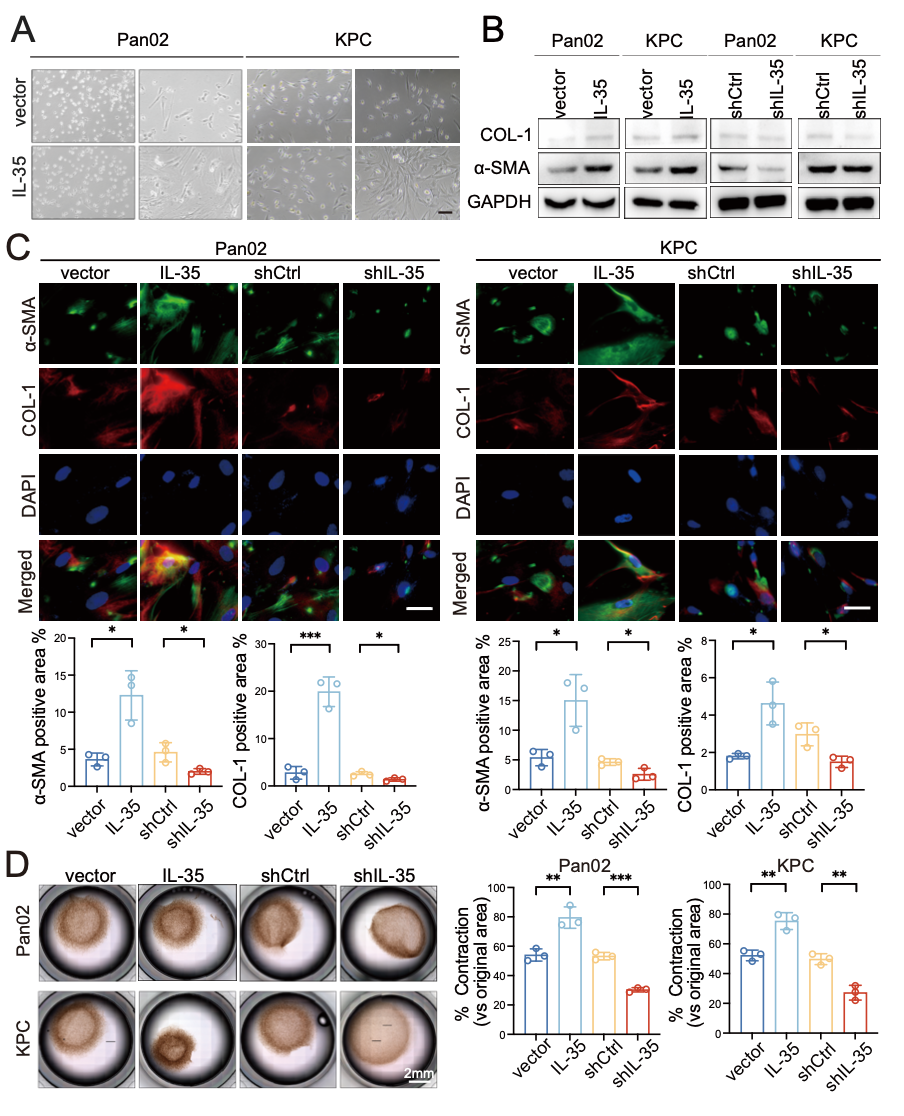


**Figure S4. IL-35 overexpression in tumor cells induces pronounced activation of PSCs.** Supernatants from indicated PDAC cells with or without IL-35 up-regulation/down-regulation were employed as CM, and mouse primary PSCs were treated with different CM for 72 hours. **A,** Morphological changes in each PSC group, represented by images. **B,** Western blotting analysis of the activation marker of PSCs. **C,** Immunofluorescence staining of activation markers in PSCs, shown with representative images. **D,** Collagen gel containing primary PSCs treated with different CM. Representative collagen gel images are displayed (7Day), and data were analyzed using an unpaired t test. Experiments above were repeated three times independently. Shown are mean ± SD; * *p* < .05. ** *p* < .01. *** *p* < .001. Scale bars, 100 μm.


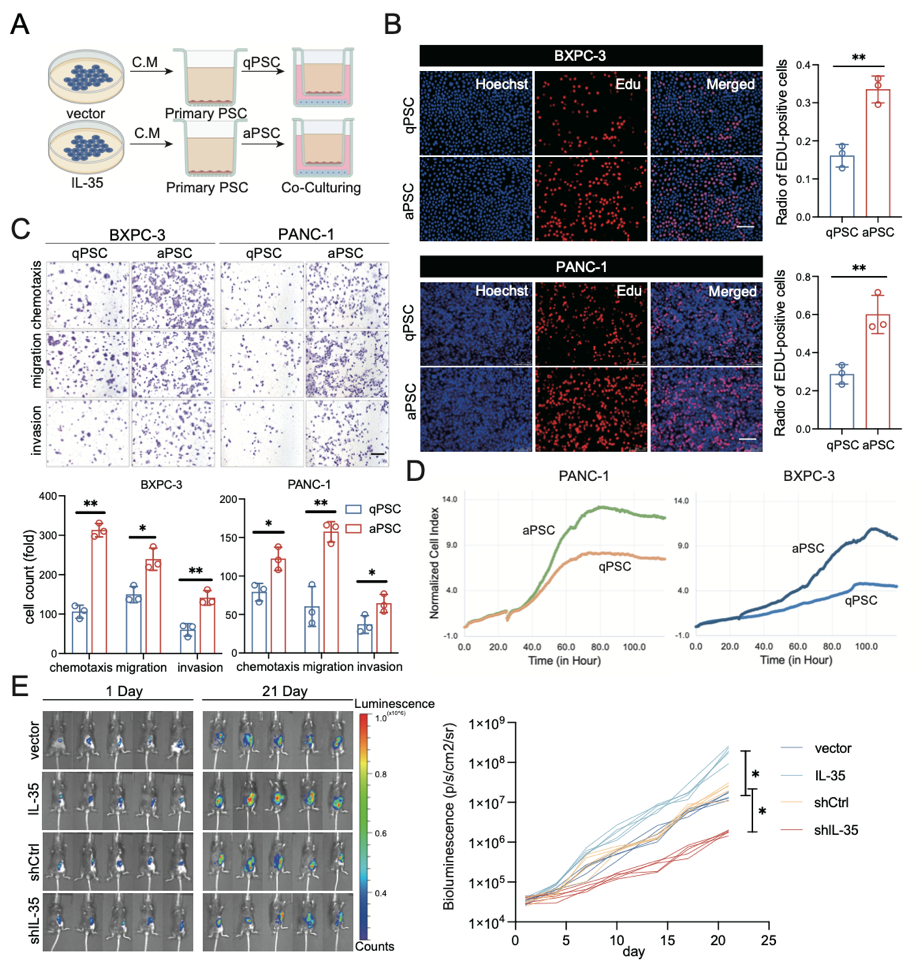


**Figure S5. Activated PSCs stimulate tumor Cell proliferation and migration. A,** Schematic diagram of cell co-culture. Supernatants from indicated PDAC cells with or without IL-35 up-regulated were used as CM. Human primary PSCs were treated with different CM for 72h. PSCs treated with vector.CM are considered quiescent PSCs (qPSC), while PSCs treated with IL-35.CM are considered activated PSCs (aPSC). qPSCs and aPSCs were co-cultured with BXPC-3/PANC-1 cells for 48h. **B,** Representative images of EdU proliferation analysis in indicated groups. Data were analyzed using unpaired t test. **C-D,** Supernatants from qPSC and aPSC were used as CM, then, BXPC-3 or PANC-1 cells were treated with different CM. Migration, invasion, and chemotaxis abilities of each group were evaluated using the transwell method. Representative images and statistical analyses of each group are shown in **C**. The cell index of the indicated group was detected by Real-Time Cell Analysis (RTCA) system **(D).** Experiments above were repeated three times independently. **E,** 1x10^5^ indicated cells were orthotopically injected into five-week-old C57BL/6 mice. Tumors were measured by IVIS weekly. Representative images of IVIS analysis for the indicated tumor model are shown. Data were analyzed using One-way ANOVA. Shown are mean ± SD; **p* < .05. ** *p* < .01. *** *p* < .001. Scale bars, 100 μm.


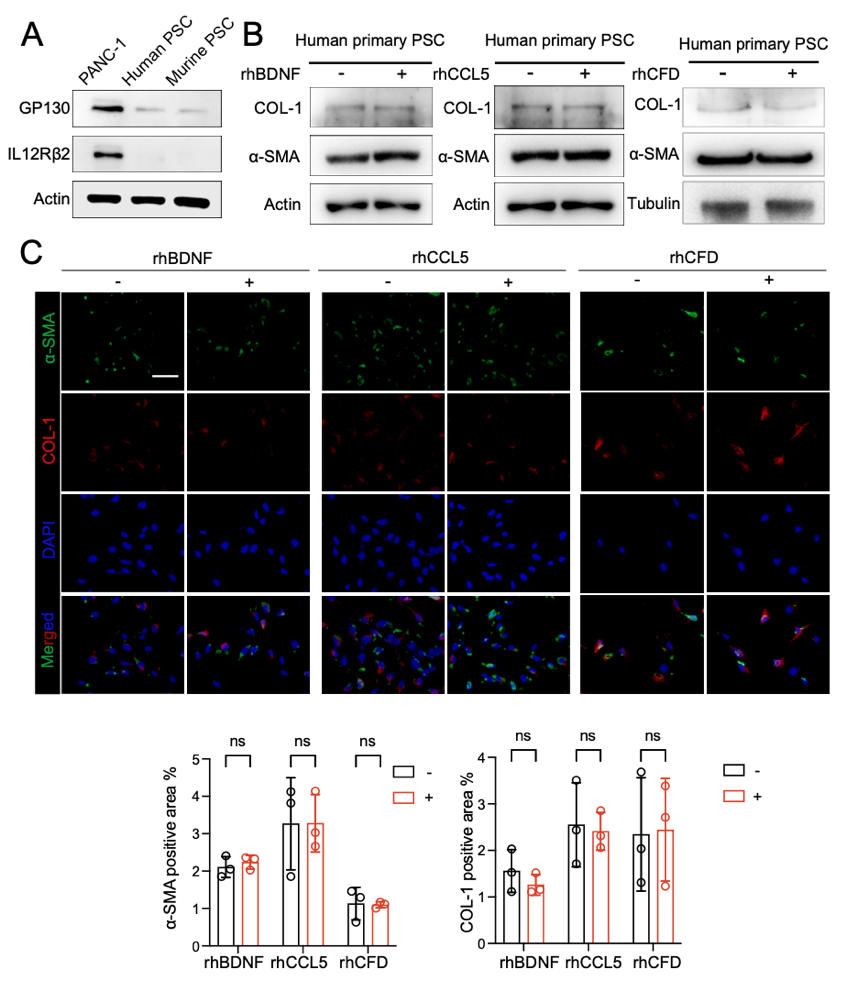


**Figure S6. BDNF, CCL5, and CFD fail to activate PSCs. A,** The expression level of GP130 and IL12Rβ2 in PANC-1, Human PSC and Murine PSC. **B-C,** The isolated human primary PSCs were treated with or without BDNF/CFD/CCL5 recombinant protein (100ng/mL) for 72h. **B,** Western blotting was used to detect the activation marker of PSCs. **C,** Representative images of immunofluorescence staining of activation markers in PSCs. Data were analyzed using an unpaired t test. Experiments above were repeated three times independently. Shown are mean ± SD; ns. no significance.


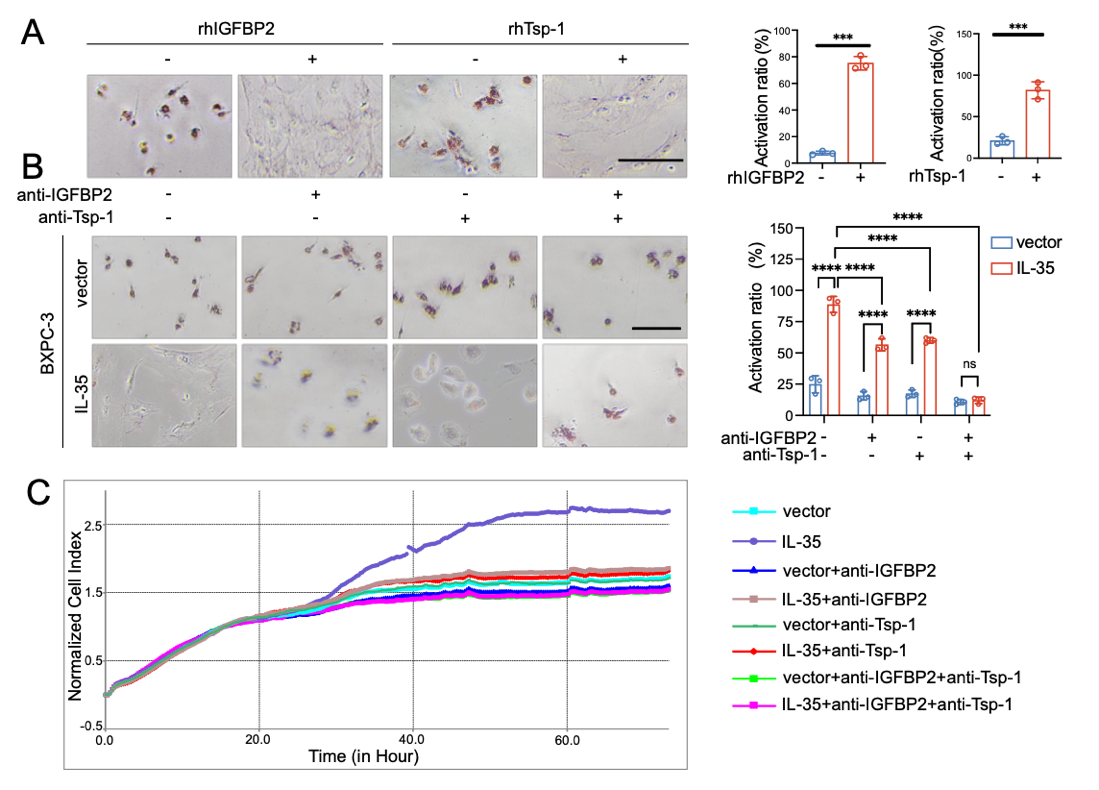


**Figure S7. Human IGFBP2 and TSP-1 are key molecules in the activation of PSC by IL-35. A,** The human primary PSCs were either treated with or without the recombinant protein IGFBP2/Tsp-1 (rhIGFBP2/Tsp-1) (100ng/mL) for 72h. Representative images of oil red o staining of PSCs are shown and the activation ratio was counted. Data were analyzed using unpaired t test. **B-C,** Supernatants from PANC-1 cells with or without IL-35 up-regulated were used as the CM. Then, the CM was treated with IGFBP2 (50ng/mL) or Tsp-1 neutralizing antibodies (100ng/mL) for 1 hour, followed by co-culturing with primary PSCs for 72h. Representative images of oil red o staining of PSCs are shown, and the activation ratio was counted **(B)**. Data were analyzed using unpaired t test. Experiments above were repeated three times independently. **C,** PSCs of indicated group were detected by RTCA system. Shown are mean ± SD; **p* < .05. ***p* < .01. ****p* < .001. Scale bars, 100 μm.


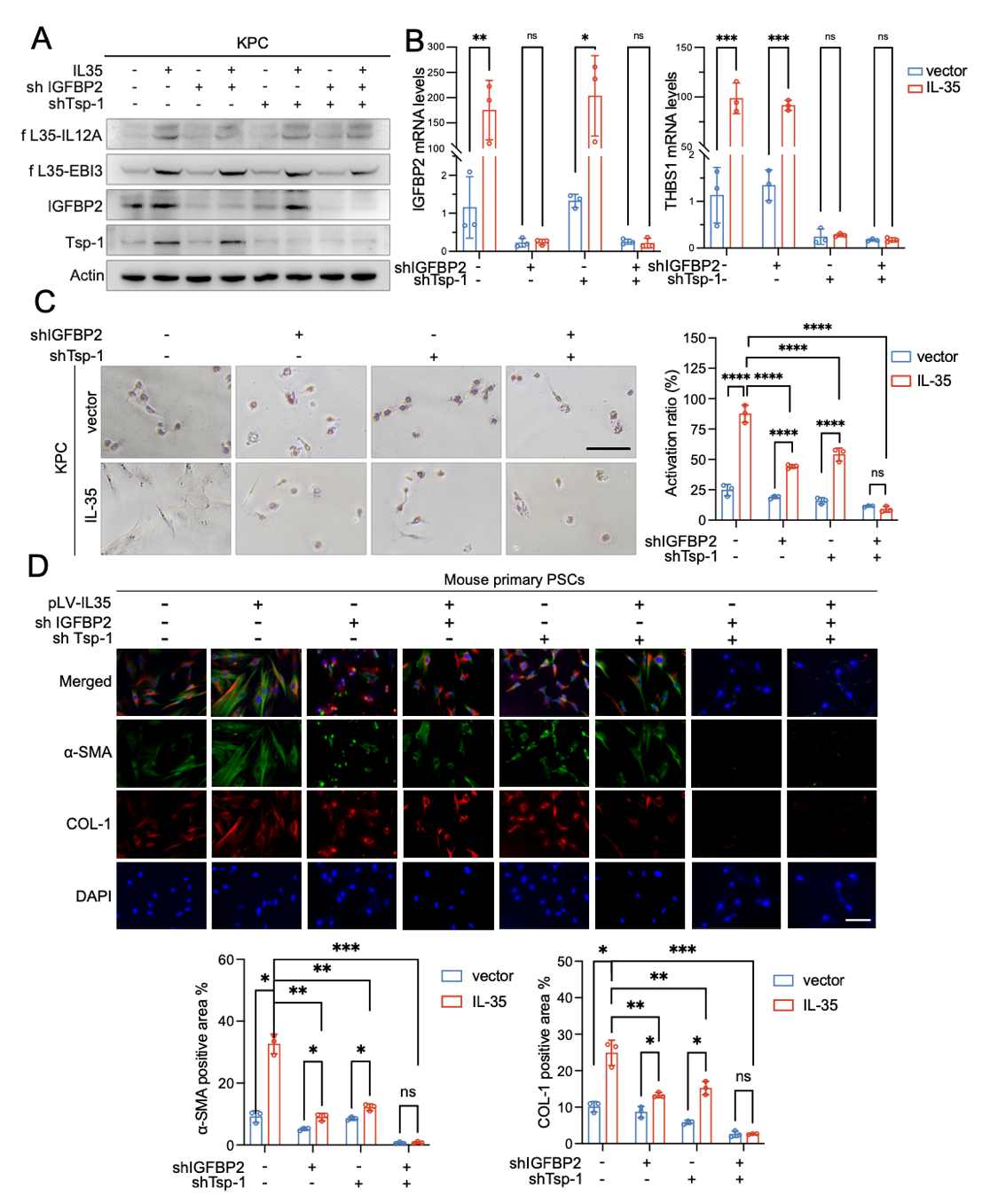


**Figure S8. Mouse IGFBP2 and Tsp-1 are key molecules in the activation of PSC by IL-35. A,** Western blot on IL-35, IGFBP2 and Tsp-1 were analyzed in indicated cell lines. **B,** Quantification of *IGFBP2* and *THBS1* levels normalized to β –actin detected by Q-PCR. **C-D,** Supernatants from indicated KPC cells were used as the CM, followed by co-culturing with mouse primary PSCs for 72h. Representative images of oil red o staining of PSCs are shown and the activation ratio was counted **(C)**. Data were analyzed using unpaired t test. Representative images of immunofluorescence staining of activation markers in PSCs **(D)**. Data were analyzed using unpaired t test. Experiments above were repeated three times independently. Shown are mean ± SD; * *p*< .05. ** *p* < .01. *** *p* < .001. Scale bars, 100 μm.


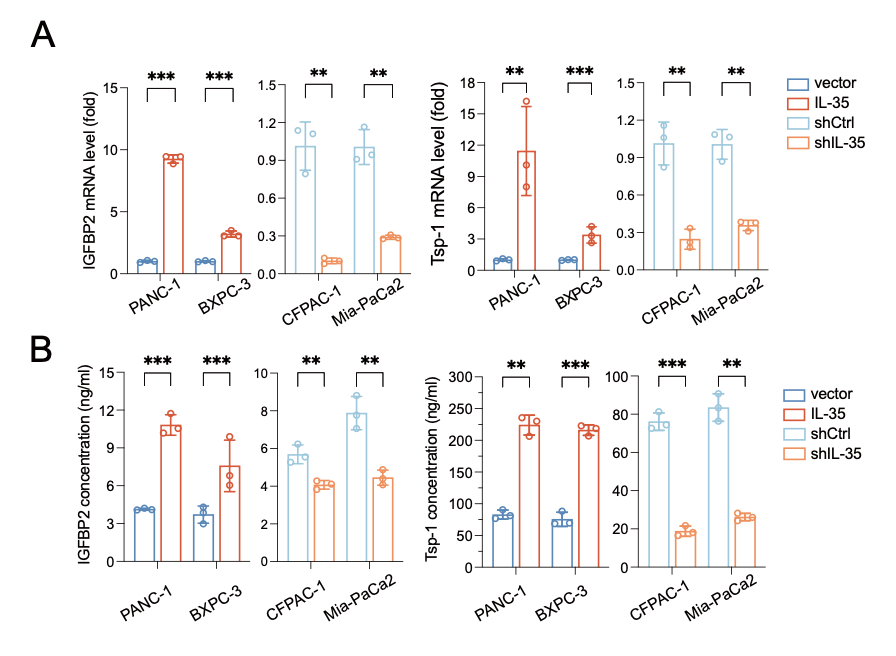


**Figure S9. Regulatory effects of IL-35 on IGFBP2 and Tsp-1. A-B,** The indicated cells were subjected to RT-PCR assays **(A)**, and ELISA **(B)** to verify the regulatory effects of IL-35 on IGFBP2 and Tsp-1. Data were analyzed using unpaired t test. Experiments above were repeated three times independently. Shown are mean ± SD; ** *p* < .01. *** *p* < .001.


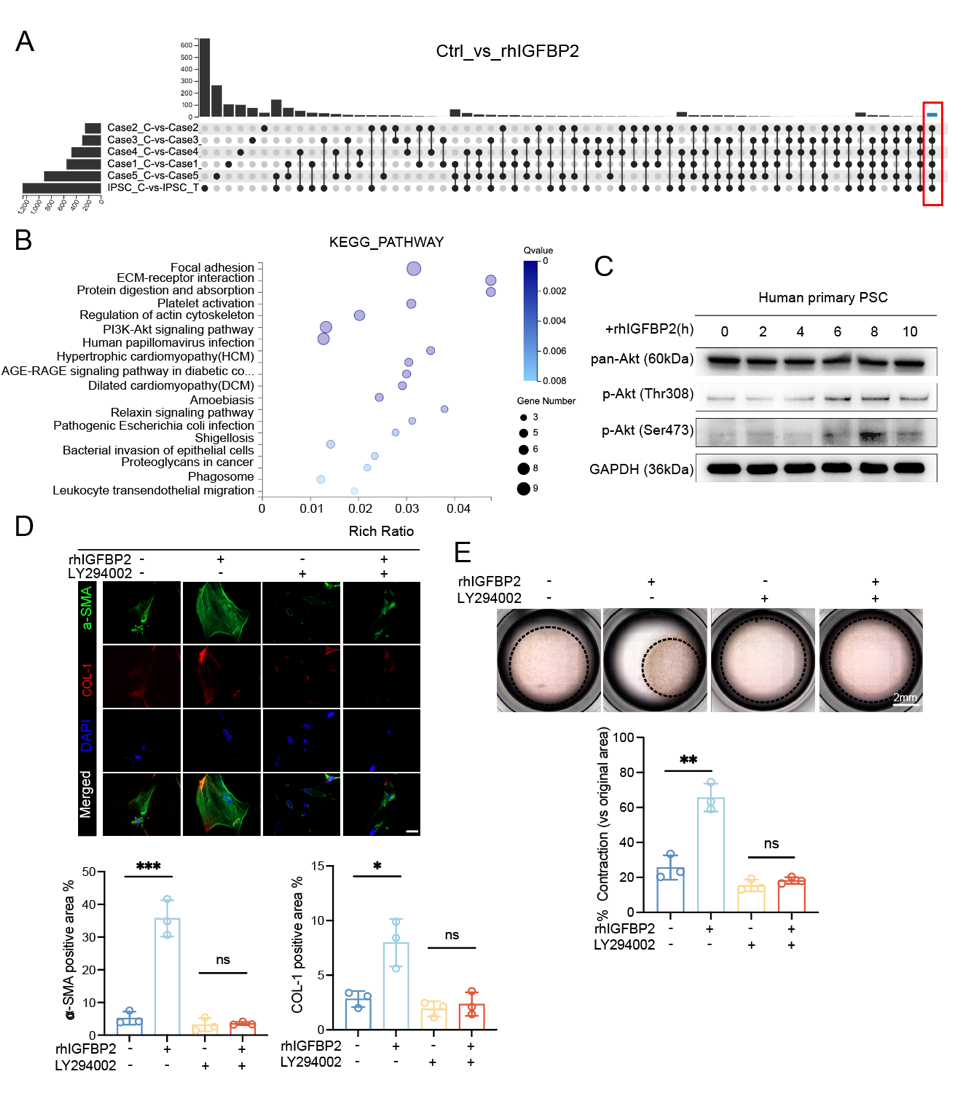


**Figure S10. IGFBP2 activates PSC through the PI3K-Akt signaling pathway.** RNA-seq was performed on Human primary PSCs from five different patients and an immortalized PSC cell line, which were treated with or without the recombinant protein IGFBP2 (100ng/mL) for 72h. **A,** Venn diagram illustrates the differentially expressed genes between the rhIGFBP2 treated group and the control group. **B,** KEGG pathway enrichment analysis was conducted on six commonly differentially expressed genes, and the results are shown. **C,** Western bloting were analyzed to detect the activation of the PI3K-Akt pathway for different time intervals. **D-E,** Human primary PSCs were pretreated with or without LY294002 (0.5μM, a PI3K inhibitor) for 2h, and then treated with rhIGFBP2 (100ng/mL) for 72h (D) or 7 days (E). Representative images of immunofluorescence staining **(D)** of activation markers in PSCs and collagen gel images **(E)** are shown. Data were analyzed using unpaired t test. Experiments above were repeated three times independently. Shown are mean ± SD; * *p* < .05. ** *p* < .01. *** *p* < .001. Scale bars, 100 μm.


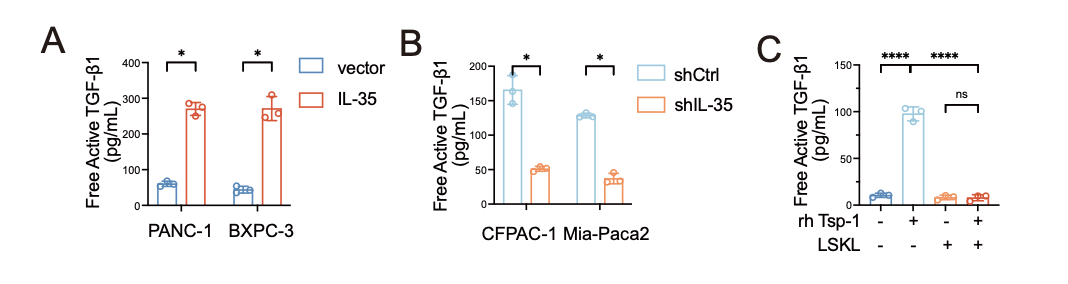


**Figure S11. Tsp-1 activate the latent TGF-β1. A-B,** The levels of free active TGF-β1 in the cell culture supernatants of each group were detected by ELISA. **C,** The levels of free active TGF-β1 under different treatment conditions were detected by ELISA. Data were analyzed using unpaired t test. Experiments above were repeated three times independently. Shown are mean ± SD; * *p* < .05. **** *p* < .0001. ns no significance.


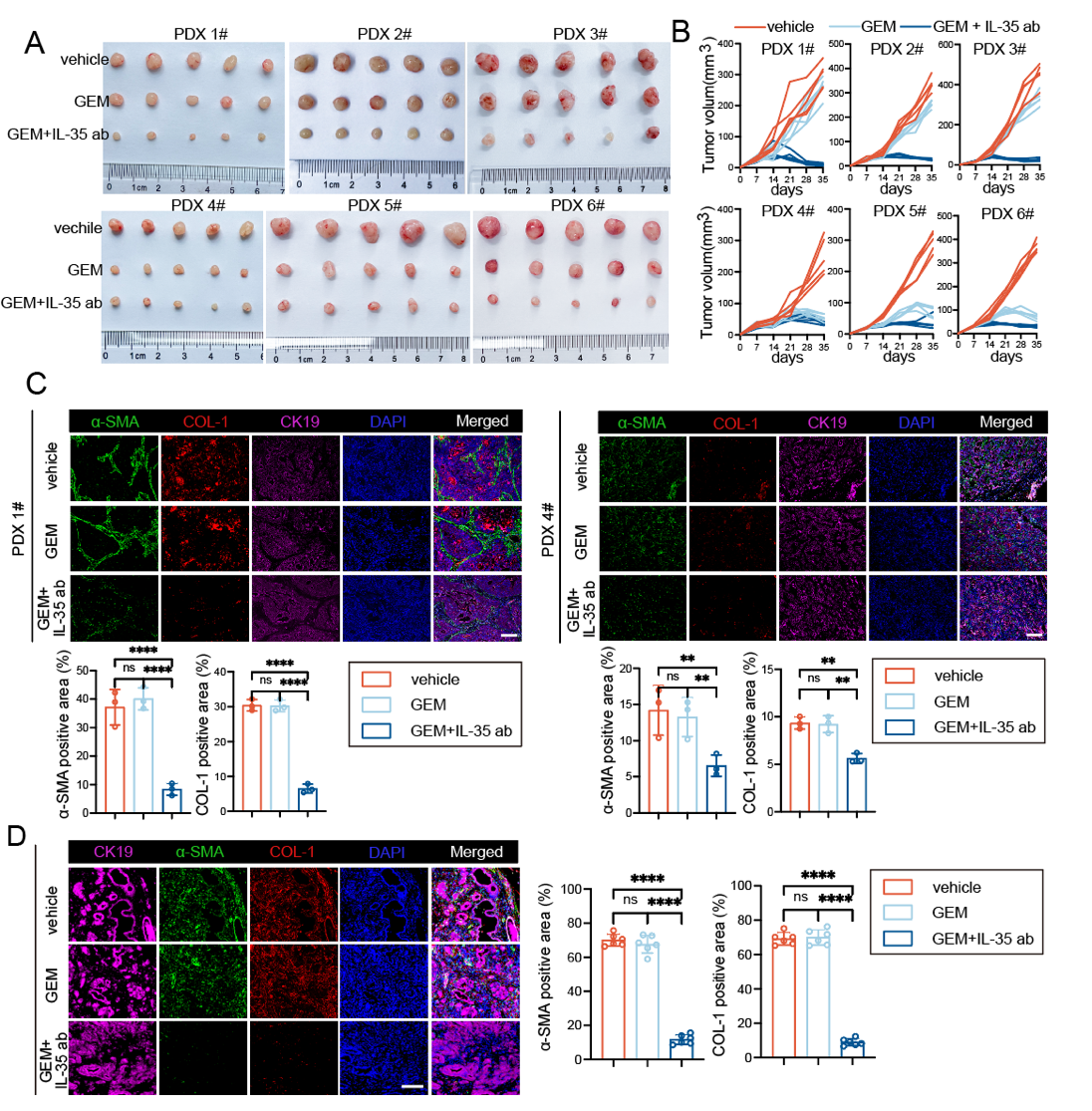


**Figure S12. Combining GEM with anti-IL-35 can effectively suppress the advancement of pancreatic cancer. A,** Representative tumor images of PDX tumor treated with combined therapy. **B,** The growth rates are shown. **C,** Sectioning of the indicated PDX tumor after embedding in paraffin was performed for Multiplex fluorescent IHC staining of CK19, α-SMA and COL-1 in tumor tissues. The representative images from indicated group (above) and the positive of α-SMA / COL-1 (below) are shown. **D,** KPC mice were administered different treatment regimens, including vehicle, GEM alone, or a combination of GEM and anti-IL-35. Sectioning of indicated KPC pancreatic tumor after embedding in paraffin was performed for Multiplex fluorescent IHC staining of CK19, α-SMA and COL-1 in tumor tissues. The representative images from indicated group (left) and the positive of α-SMA / COL-1 (right) are shown. Data were analyzed using unpaired t test. Shown are mean ± SD; * *p* < .05. ** *p* < .01. *** *p* < .001. **** *p* < .0001. ns no significance. Scale bars, 100 μm.


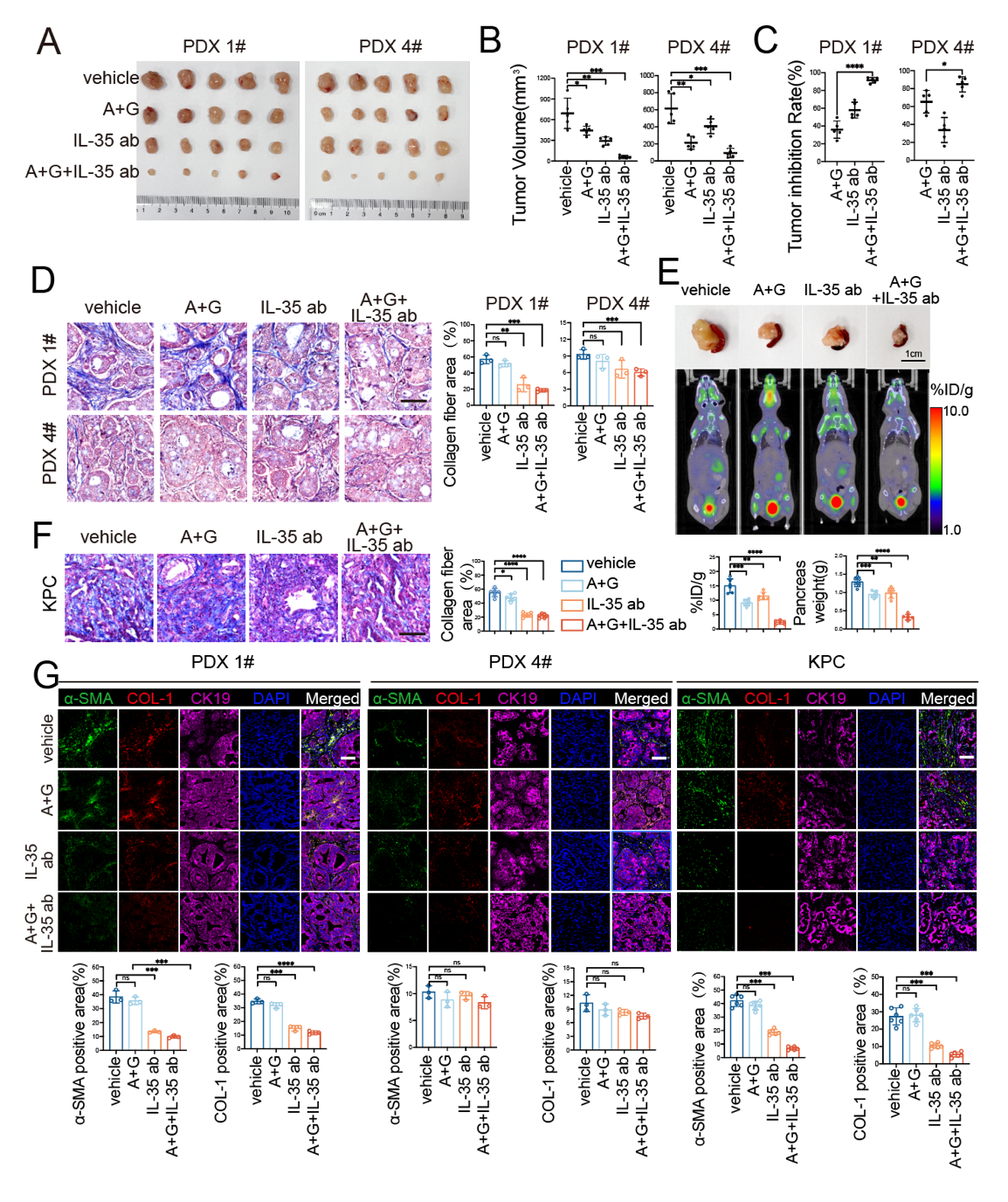


**Figure S13. Anti-IL-35 efficiently diminishes fibrosis in PDAC while improving the effectiveness of A+G treatment.** **A-B,** Representative tumor images of PDX tumor treated with combined therapy and tumor volums. **C,** Tumor inhibition rate of various treatment groups in PDX tumor compared to the control group. **D,** Sectioning of the indicated PDX tumor after embedding in paraffin was performed for Masson trichrome staining to determine the degree of tissue fibrosis. Representative images (left) and collagen fiber percentage (right) were shown. **E,** KPC mice were administered different treatment regimens, including saline vehicle, A+G, or a combination of A+G and anti-IL-35 (25μg) twice a week. The effects of these treatments on pancreatic tumors in KPC mice were evaluated using macroscopic imaging and tumor burden measurement using PET-CT scans after 14 days of treatment. Scale. Representative images of these assessments were provided. **F,** Sectioning of the indicated KPC pancreatic tumor after embedding in paraffin was performed for Masson trichrome staining to determine the degree of tissue fibrosis. Representative images (left) and collagen fiber percentage (right) were shown. **G,** Sectioning of the indicated PDX tumors and KPC tumors after embedding in paraffin was performed for Multiplex fluorescent IHC staining of CK19, α-SMA and COL-1 in tumor tissues. The representative images from indicated group (above) and the positive of α-SMA / COL-1 (below) are shown. Data were analyzed using unpaired t-test. Shown are mean ± SD; * *p* < .05. ** *p* < .01. *** *p* < .001. **** *p* < .0001. ns no significance. Scale bars, 100 μm.


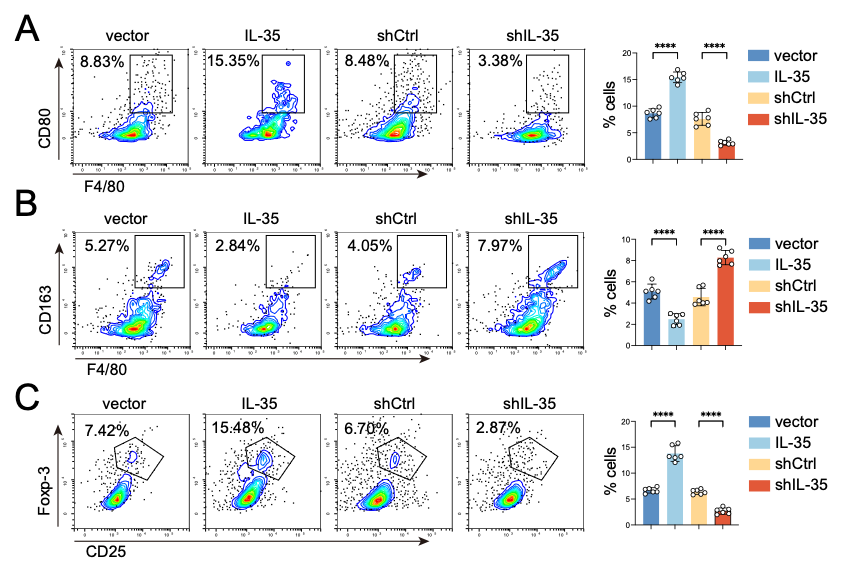


**Figure S14. Effects of IL-35 on the immune microenvironment in PDAC.** **A,** Frequency of M1-like macrophages in orthotopic tumors derived from KPC cells with IL-35 overexpression, knockdown, or control, quantified by flow cytometry. Data are presented as representative flow cytometry plots and summarized bar graphs. **B,** Proportion of M2-like macrophages under the same conditions. **C,** Percentage of regulatory T cells in the tumor microenvironment. Data represent mean ± SD from n = 6 mice per group; statistical significance was assessed using one-way ANOVA followed by Tukey’s post-hoc test.


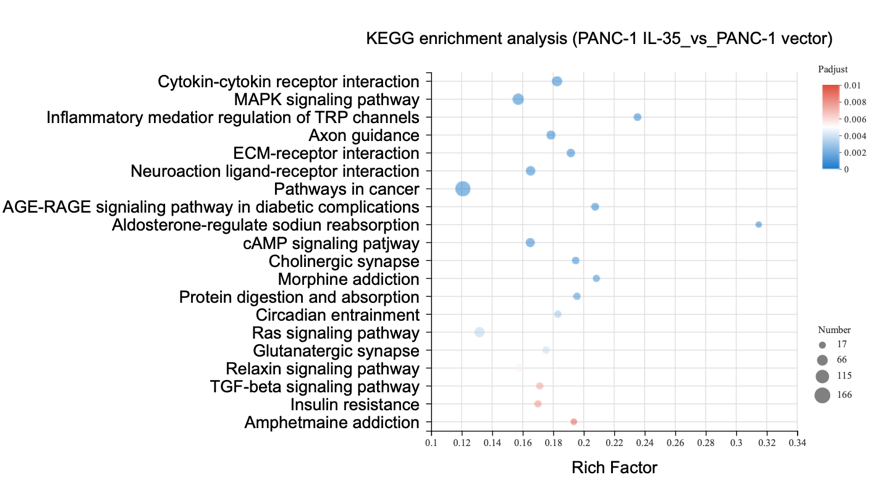


**Figure S15. RNA-seq pathway enrichment analysis of IL-35–stimulated PANC-1 cells.** KEGG enrichment analysis was performed on RNA-seq data from PANC-1 cells.
