# Supplementary material for: Tumor‐Derived Interleukin 35 Promotes Fibrosis in the Tumor Microenvironment of Pancreatic Cancer by Activating Pancreatic Stellate Cells
Source: Adv Sci (Weinh). 2025 Nov 14;13(5):e09074. doi: 10.1002/advs.202509074 (PMC12850332; doi:10.1002/advs.202509074)
Supplement: Supplementary file 2 — Supporting Information [file ADVS-13-e09074-s003.docx]

Supplementary Table 1. Detailed information of antibodies for IHC/IF and WB used in this study.

| Name | Source | Concentration | Cat. No. | RRID |
| --- | --- | --- | --- | --- |
| anti-EBI3 antibody | Abcam | 1:10000 (WB) | ab124694 | AB_10972489 |
| anti-EBI3 antibody | Abcam | 1:400 (IHC) | ab124694 | AB_10972489 |
| anti-IL-12A antibody | Abcam | 1:1000 (WB) | ab131309 |  |
| anti-IL-12A antibody | Abcam | 1:200 (IHC) | ab131309 |  |
| anti-αSMA antibody | Abcam | 1:1000 (WB) | ab124964 | AB_10972489 |
| anti-αSMA antibody | Abcam | 1:400 (IHC/IF) | ab124964 | AB_10972489 |
| anti-Collagen 1 antibody | Abcam | 1:500 (WB) | ab316223 |  |
| anti-Collagen 1 antibody | Abcam | 1:100 (IHC/IF) | ab316223 |  |
| anti-Cytokeratin 19 antibody | Abcam | 1:1000 (IHC) | ab52625 | AB_2281020 |
| anti-GAPDH antibody | Abcam | 1:10000 (WB) | ab8245 | AB_2107448 |
| anti-beta Tubulin | Abcam | 1:10000 (WB) | ab6046 | AB_2210370 |
| anti-beta Actin | Abcam | 1:10000 (WB) | ab8226 | AB_306371 |
| Thrombospondin-1 Rabbit mAb | Cell Signaling Technology | 1:1000 (WB) | #37879S | AB_2799123 |
| anti-Thrombospondin 1 antibody | Abcam | 1:100 (IHC) | ab1823 | AB_2201948 |
| anti-IGFBP2 antibody | Abcam | 1:2000 (WB) | ab188200 | AB_2938998 |
| anti-IGFBP2 antibody | Abcam | 1:1000 (IHC) | ab188200 | AB_2938998 |
| Stat1 Antibody | Cell Signaling Technology | 1:1000 (WB) | #9172S | AB_2198300 |
| Stat4 Rabbit mAb | Cell Signaling Technology | 1:1000 (WB) | #2653S | AB_2255156 |
| Phospho-Stat1 Rabbit mAb | Cell Signaling Technology | 1:1000 (WB) | #9167S | AB_561284 |
| Phospho-Stat4 Antibody | Cell Signaling Technology | 1:1000 (WB) | #5267S | AB_10545446 |
| IGF-I Receptor β Rabbit mAb | Cell Signaling Technology | 1:500 (WB) | #9750 | AB_10950969 |
| Phospho-IGF-IR β Antibody | Cell Signaling Technology | 1:500 (WB) | #3021 | AB_331578 |
| Akt (pan) Rabbit mAb | Cell Signaling Technology | 1:2000 (WB) | #4691 | AB_915783 |
| Phospho-Akt (Ser473) Rabbit mAb | Cell Signaling Technology | 1:1000 (WB) | #4060 | AB_2315049 |
| Phospho-Akt (Thr308) Rabbit mAb | Cell Signaling Technology | 1:1000 (WB) | #13038 | AB_2629447 |
| SMAD2/3 Rabbit mAb | Cell Signaling Technology | 1:1000 (WB) | #8685 | AB_10889933 |
| Phospho-SMAD2 Rabbit mAb | Cell Signaling Technology | 1:500 (WB) | #3108 | AB_490941 |
| Phospho-SMAD3 Rabbit mAb | Cell Signaling Technology | 1:500 (WB) | #9520 | AB_2193207 |

Supplementary Table 2. Detailed information of Reagents used in this study.

| Name | Source | Cat.No. |
| --- | --- | --- |
| Modified Masson's Trichrome Stain Kit | Solarbio | G1346 |
| Oil red O | Sigma | O9755 |
| Collagen Type 1 | Corning | 356236 |
| EdU Proliferation Kit (iFluor 647) | Abcam | ab222421 |
| Proteome Profiler Human XL Cytokine Array Kit | R&D Systems | ARY022B |
| EZ-ChIP | millipore | 17-371 |
| Dual-Luciferase Reporter Assay System | Promega | E1910 |
| IL-35 EBI3 Antibody | Shenandoah | 210-301-C56S |
| anti-Human EBI3 neutralizing antibody | R&D Systems | AF2526 |
| Human IGFBP-2 neutralizing antibody | R&D Systems | AF674 |
| Thrombospondin 1 neutralizing antibody | Thermo Fisher Scientific | 39-9300 |

Supplementary Table 3. Detailed information of primers used in this study.

| Gene Name | Forward | Reverse |
| --- | --- | --- |
| IGFBP2 | TGCAACAATGGCGATGACCAC | CCGTTCAGAGACATCTTGCACT |
| THBS1 | TGCCCCTTGGAACACAATCCG | TCCAGCATAGTCATCGTCCCT |
| IGFBP2-BM1 (ChIP) | TCCAAGACCCCTGCAACTGA | CGCCCGCTAAAATAATCCCT |
| IGFBP2-BM2 (ChIP) | CTTACCAAAGTCTTTCCCGAT | CTCTTTCTGACTTCAAGGCTC |
| THBS1-BM1 (ChIP) | TGCCACCGTATAGGTCACCTT | ACAGAGTAGAAACCAGCACT |
| THBS1-BM2 (ChIP) | CTGGATCACAGCAGAGGGAGC | ATTGCCATGAGTGCCGCTG |

Supplementary Table 4. Detailed information of recombinant proteins and inhibitors.

| Name | Source | Cat.No. |
| --- | --- | --- |
| Recombinant Human IL-35 Protein | R&D Systems | 8608-IL |
| Recombinant protein of human BDNF | OriGene Technologies | TP317124M |
| Recombinant Human CCL5 Protein | R&D Systems | 335-RM-025 |
| Factor D (CFD) Human Recombinant Protein | OriGene Technologies | TP309272M |
| IGFBP2 Human Recombinant Protein | OriGene Technologies | TP302573M |
| Tsp-1 Human Recombinant Protein | OriGene Technologies | TP314802M |
| Picropodophyllin | MCE | HY-15494 |
| LSKL, Inhibitor of Thrombospondin (TSP-1) | MCE | HY-P0299 |
| LY294002 | MCE | HY-10108 |

Supplementary Table 4. Detailed information of shRNA oligos sequences.

| sh-Human IL-12A | AAAAGAGACCTCTTTCATAACTATTGGATCCAATAGTTATGAAAGAGGTCTC |
| --- | --- |
| sh-Human EBI3 | AAAATCACGGATGTCCAGCTGTTTTGGATCCAAAACAGCTGGACATCCGT  GA |
| sh-mouse IL-12A | AAAATGTCCAAGCTGCTCTTCCTGTCACTTGCCTTGGATCCAAGGCAAGT  GACAGGAAGAGCAGCTTGGACA |
| sh-mouse EBI3 | AAAAGGTCCAGCATGTGTCAATCACGCTACCTCTTGGATCCAAGAGGTAG  CGTGATTGACACATGCTGGACC |
| sh-mouse IGFBP2 | AAAAGCATGGCCGGTACAACCTTAATTCGTTAAGGTTGTACCGGCCATGC |
| sh-mouse THBS1 | AAAAGCTGGAAAGATTTCACTGCATTTGGATCCAAATGCAGTGAAATCTTT  CCAGC |

Supplementary Table 5: Detailed information of patients whose specimens were used for establishment of PDX in vivo PDX mouse models.

| Patient ID | Age | Gender | TNM stage | Pathological stage | PDAC differentiation grade |
| --- | --- | --- | --- | --- | --- |
| PDX 1# | 66 | Male | T2N0M0 | IB | moderated |
| PDX 2# | 54 | Male | T2N0M0 | IIB | moderated |
| PDX 3# | 59 | Male | T2N0M0 | IB | moderated |
| PDX 4# | 54 | Female | T2N0M0 | IB | moderated |
| PDX 5# | 65 | Male | T2N0M0 | IIB | moderated |
| PDX 6# | 61 | Male | T2N0M0 | IB | moderated |
